# An electroporation-free method based on Red recombineering for markerless deletion and genomic replacement in the *Escherichia coli* DH1 genome

**DOI:** 10.1371/journal.pone.0186891

**Published:** 2017-10-24

**Authors:** Yanlong Wei, Pingping Deng, Ali Mohsin, Yan Yang, Huayan Zhou, Meijin Guo, Hongqing Fang

**Affiliations:** 1 State Key Laboratory of Bioreactor Engineering, East China University of Science and Technology, Shanghai, China; 2 Institute of Health Sciences, Anhui University, Economic and Technology Development Zone, Hefei, Anhui, China; 3 Luoyang Vocational & Technical College, Luoyang, Henan, China; 4 Institute of Biotechnology, Academy of Military Medical Sciences, Beijing, China; Tulane University Health Sciences Center, UNITED STATES

## Abstract

The λ-Red recombination system is a popular method for gene editing. However, its applications are limited due to restricted electroporation of DNA fragments. Here, we present an electroporation-free λ-Red recombination method in which target DNA fragments are excised by I-CreI endonuclease *in vivo* from the landing pad plasmid. Subsequently, the I-SceI endonuclease-cutting chromosome and DNA double-strand break repair were required. Markerless deletion and genomic replacement were successfully accomplished by this novel approach. Eight nonessential regions of 2.4–104.4 kb in the *Escherichia coli* DH1 genome were deleted separately with selection efficiencies of 5.3–100%. Additionally, the recombination efficiencies were 2.5–45%, representing an order of magnitude improvement over the electroporation method. For example, for genomic replacement, lycopene expression flux (3.5 kb) was efficiently and precisely integrated into the chromosome, accompanied by replacement of nonessential regions separately into four differently oriented loci. The lycopene production level varied approximately by 5- and 10-fold, corresponding to the integrated position and expression direction, respectively, in the *E*. *coli* chromosome.

## Introduction

Chromosomal modifications are critical tools for genomic and metabolic engineering [1–4]. In *Escherichia coli*, the λ-Red recombination system can be used for chromosomal modifications, including gene deletions, mutations, and integration. This system is constituted by three proteins: Exo, Gam, and Beta. Exo protein binds to the end of linear DNA and generates 3′ overhangs, whereas Gam binds to the RecBCD complex, thereby preventing the degradation of double-stranded DNA. Beta mediates annealing between complementary strands [5–7].

Based on Red recombineering, many approaches have been established for genomic markerless deletion through electroporation into cells with polymerase chain reaction (PCR) fragments, including selection markers, such as the chloramphenicol (Cm)-resistance gene SacB box and I-SceI site [8–11]. However, the electroporation method is limited by its very low efficiency; only a small percentage of cells can accept target DNA fragments introduced by electroporation [12]. Moreover, Lee et al achieved lower electroporation efficiency in pathogenic *E*. *coli* strains compared with that in MG1655 [13]. Thus, not all species or *E*. *coli* mutants are readily transformed with linear DNA fragments.

Another method for deletion is supply of target DNA by a donor plasmid that can then be cleaved by an endonuclease *in vivo*, eliminating the need for electroporation of DNA fragments into cells. “Gene gorging” is based on the employment of a donor plasmid, excised by the I-SceI endonuclease, to yield donor fragments in living cells [12]; however, the low efficiency of recombination in pathogenic *E*. *coli* strains limits the universal application of this method [13]. Thus, Lee et al. [13] developed a high efficiency method, called “Gene Doctoring,” which leaves an 80-bp DNA “scar,” thus limiting the repeated use of recombineering in the same bacterium. Kuhlman and Cox [14] described a two-step λ-Red system that permitted integration of a 7-kb fragment into the target location. In this method, donor fragments are generated *in vivo*; however, the fragments containing the landing pad (the DNA sequence used as a homologous region in the second recombination step flanking the antibiotic gene cassette) had to be electroporated into cells. Recently, Tas et al [15] developed an integrated system based on a series of plasmids that could precisely modify the genome of *E*. *coli*; however, this system is limited by the use of a heavy metal, i.e., nickel chloride, to achieve negative selection against *tetA* expression, eventually causing adverse effects on the environment and increasing health hazards.

Therefore, in this study, we aimed to establish an electroporation-free method for genomic modification of *E*. *coli* and to overcome the shortcomings of existing methods simultaneously, using a landing pad plasmid containing I-SceI and I-CreI sites for successive DNA double-strand cleavage to deliver an antibiotic cassette for entry and exit from the *E*. *coli* chromosome.

## Materials and methods

### Strains, plasmids, and reagents

The strains and plasmids used in this study are described in S1 Table. *E*. *coli* DH5α and *E*. *coli* DH1 were used for plasmid construction and chromosomal modification, respectively. Plasmids pKOBEG and pKOBEGA were acquired from Christophe d'Enfert [5]. Gene *I-CreI* and *I-SceI* were artificially synthesized by GENEWIZ Company and cloned into the *Kpn*I/*BamH*I site of pUC19 to generate pUCIS and pUCIC. The sequences of *I-SceI* and *I-CreI* are described in S2 Table. The commercial plasmid pET28a was purchased from EMD Biosciences (Novagen). Plasmids pKOBEGK, pSNA, pSNK, and pCNA were derived from pKOBEG or pKOBEGA, and plasmid maps of all plasmids containing the λ-Red system or endonuclease gene used in this study are shown in S1 Fig. The construction process for pKOBEGK, pSNA, pSNK, and pCNA is shown in S2 Fig. The Rapid DNA Ligation Kit, DNA polymerase, Gel Extraction Kit, and Plasmid Miniprep Kit were purchased from Thermo Fisher Scientific Inc. (MA, USA), TaKaRa Biotechnology Co. (Dalian, China), or Corning Inc. (Wujiang, China). All assays were performed according to the manufacturer’s instructions.

### Cell culture

*E*. *coli* DH5α was grown in tubes containing 5 mL Luria Bertani (LB) medium at 37°C. Cells containing helper plasmid (pCNA or pSNK) were grown at 30°C, and for helper plasmid curing, these cells were incubated at 42°C. LBS agar plates (LB agar plates without NaCl but containing 6% sucrose) were used for *sacB* counter-selection. For lycopene production, single colonies were picked, placed into tubes containing 5 mL LB medium, and cultured overnight at 30°C with shaking at 220 rpm. Next, the overnight culture was diluted into 5 mL FMGT medium (per 600 mL: 9 g tryptone, 7.2 g yeast extract, 1.8 g NaH_2_PO_4_·2H_2_O, 4.2 g K_2_HPO_4_·3H_2_O, 1.5 g NaCl, 3 g Tween 80, 6 g glycerol, 0.3 g MgSO_4_, 1.2 g glucose), resulting in an original inoculum concentration of 0.05 (OD_600nm_). The strains were then grown under the same conditions for 24 h, and lycopene was then extracted for further evaluation. Ampicillin (Amp, 50 μg/mL), Cm (25 μg/mL), kanamycin (Kan, 25 μg/mL), and tetracycline (Tc, 10 μg/mL) were added as required.

### Measurement of lycopene production

Extraction of lycopene from *E*. *coli* was carried out as described previously [16]. The *E*. *coli* cultures were centrifuged at 11,340 × *g* for 3 min and washed once with water. Samples were extracted with acetone in the dark at 55°C for 15 min. To quantify lycopene production, a spectrophotometric method was employed at a wavelength of 474 nm (UV-2100 Spectrophotometer; UNICO Company, Shanghai, China). Lycopene (Sigma-Aldrich) was used as the standard. The results represent the means ± standard deviations of three independent experiments.

### Construction of the landing pad plasmid pBDC-Xd

**Fig 1:**
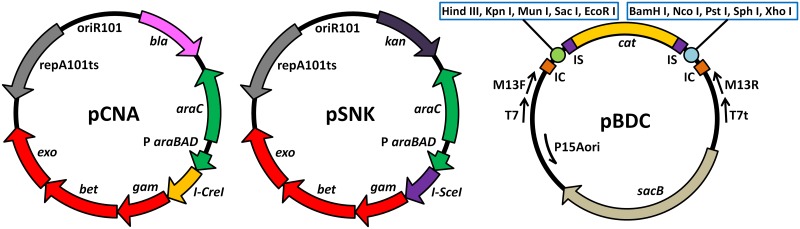
Plasmid maps of pCNA, pSNK, and pBDC. The helper plasmids pCNA and pSNK, which could be eliminated by incubation at 42°C, were obtained upon insertion of *I-CreI* and *I-SceI*, respectively, into pKOBEG. The *bla* cassette was obtained from pET3b (Novagen), and the *kan* cassette was obtained from pET28a (Novagen). The p15A ori and *cat* cassettes of the landing pad plasmid pBDC were obtained from pACYCDuet-1 (commercial plasmid purchased from EMD Biosciences). IC and IS represent the I-CreI endonuclease recognition site and I-SceI endonuclease recognition site, respectively.

The plasmid pBDC-Xd was based on pBDC (Fig 1) for deletion of *E*. *coli* DH1 genomic nonessential regions. In this study, ‘X’ in the names of strains, plasmids, fragments, and primers represents the number of nonessential regions (X = 1, 2, 5, 7, 8, 19, 55, or 63). The Cm-resistance cassette flanked by the I-SceI recognition site was amplified by PCR from pBDC with primers T7 and T7t. The 1000-bp PCR fragment was digested with *EcoR*I-*BamH*I, generating CmIS. To generate the right homology region (XR), a 500-bp PCR fragment was amplified using the primers XR-5/XR-3 from the genome of *E*. *coli* DH1 and digested with *Bam*HI-*Xho*I. Primers XL-5/XL-3d, designed according to the methods of Yu et al. [11], were used to amplify the left homology region and landing pad region (50 bp DNA sequence of the 5′-end of the right homology region) from the genome of *E*. *coli* DH1. The PCR products were digested with *Hin*dIII-*Eco*RI, generating the fragment XL. Three fragments, i.e., CmIS, XR, and XL, were cloned into the *Hin*dIII-*Xho*I site of pBDK together, generating pBDC-Xd. All primers used in this study are listed in S3 Table.

### Construction of the landing pad plasmid pBDC-Yi and counter-clockwise landing pad plasmid pBDC-Yri

**Fig 2:**
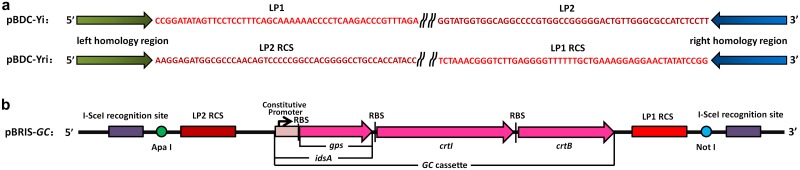
The landing pad plasmid and donor plasmid used for genomic replacement. (a) The landing pad regions of plasmids pBDC-Yi and pBDC-Yri. (b) Linearized vector map of donor plasmid pBRIS-*GC*. RCS indicates the reverse complementary sequence.

Plasmid pBDC-Yi, designed following the methods of Kuhlman and Cox [14], was used to introduce the *cat* cassette flanked by I-SceI recognition sites and two landing pad regions into the nonessential region of *E*. *coli* DH1. Here, ‘Y’ represents the number of nonessential regions as well, but for integration (Y = 8, 23, 57, or 64). The method for construction of the landing pad plasmid pBDC-Yi was similar to that for pBDC-Xd. Landing pad sequences were used as short homology regions for the second step of integration. The plasmid pBDC-Yi carried two landing pads, as shown in Fig 2a. Landing pad 1 (LP1) was 381–430 bp of plasmid pET3b, and landing pad 2 (LP2) was 788–837 bp of pET3b. LP1 was a part of primer Y-L3i at the 3′ side of fragment YLi-LP1, which was amplified by PCR from the DH1 genome with the primers Y-L5 and Y-L3i. Primer Y-R5i, containing LP2, was employed with its partner primer Y-R3 to amplify the *E*. *coli* DH1 genome, generating the right homology region flanked by landing pad sequences, YRi-LP2.

Plasmid pBDC-Yri was similar to pBDC-Yi. However, the left homology region of pBDC-Yri was flanked by the reverse complementary sequence of LP2 (LP2 RCS). The reverse complementary sequence of LP1 (LP1 RCS) was at the 5′ site of the right homology region (Fig 2a).

### Construction of the *GC* cassette and donor plasmid pBRIS-*GC*

The *GC* cassette included *idsA* from *Archaeoglobus fulgidus* DSM 4304 and *crtI-crtB* amplified from *Pantoea agglomerans* (Fig 2b). The *idsA* sequence contained the geranylgeranyl diphosphate synthase (*gps*) gene and its constitutive promoter. Thus, the *GC* cassette would be constitutively expressed without induction. The products of the *GC* cassette were *Gps*, phytoene synthase (*CrtB*), and phytoene desaturase (*CrtI*), which catalyzed the generation of geranylgeranyl diphosphate (GGPP), then phytoene, and finally lycopene from endogenous IPP. The sequence of the *GC* cassette is given in S2 Table.

The plasmid pBRIS-*GC* carries the *GC* cassette flanked by I-SceI recognition sites. Its backbone contains pBM1 ori and the Tc resistance gene from plasmid pBR322 (New England Biolabs, Inc.). The construction process is shown in S3 Fig.

### Genomic deletion and replacement

For markerless deletion, the landing pad plasmid pBDC-Xd and helper plasmid pCNA were transformed into *E*. *coli* DH1 chemically competent cells. After incubation for about 1 h in LB medium, 500 μL was plated on LB agar plates containing Amp and Cm and grown overnight at 30°C. A single colony was picked and suspended in 5 mL LB medium supplemented with Amp and Cm, followed by overnight shaking at 30°C. Next, 50 μL of overnight culture was diluted into 5 mL LB medium supplemented with Amp followed by shaking at 30°C. When the OD_600nm_ reached 0.2–0.3, l-arabinose was added to the medium at a final concentration of 0.2% to induce the expression of λ-Red proteins and I-CreI endonuclease. After induction for 4 h at 30°C, the medium was diluted 10× and then plated on LBS agar plates containing Cm to select against pBDC plasmids. After incubation at 42°C for about 16 h, the potential recombinant colonies were picked from agar plates and placed into LB medium for further confirmation by PCR using the primers X-0 and X-1 flanking the sides of the recombinant region. Positive colonies yielded a product of about 2300 bp. In addition, pCNA harbored a temperature-sensitive replication origin, which was easily controlled by growing the cells at 42°C. Later, positive colonies were plated on LB agar plates containing Amp to verify the elimination of the helper plasmid pCNA. The residual pBDC-Xd was easily verified by PCR using primers T7/T7t or M13F/M13R.

After insertion of the *cat* cassette into the nonessential region of the *E*. *coli* DH1 genome, the recombinant cells were transformed with the helper plasmid pSNK. Subsequently, after incubation for about 1 h in LB medium, cells were plated on LB agar plates containing Kan and were then incubated again for about 20 h at 30°C. Colonies were then picked, suspended in 5 mL LB medium supplemented with Kan, and incubated overnight at 30°C with shaking. Subsequently, samples were then diluted in 5 mL LB medium supplemented with Kan, followed by addition of l-arabinose when the OD_600nm_ reached 0.2–0.3. Cells were then incubated at 30°C for 4 h, after which they were diluted 100× and grown overnight on LB agar plates at 42°C. Colonies were then identified by PCR using the primers X-0 and X-1, and the product was about 1000 bp. In addition, the plasmid pSNK harboring the temperature-sensitive replication origin was easily cured by growing the cells at 42°C. Positive samples were plated on LB agar plates containing Cm and LB agar plates containing Kan to verify the elimination of the *cat* cassette and pSNK, respectively, upon sequencing with the primers X-0 and X-1.

The method for genomic replacement of nonessential regions with the lycopene expression flux was similar to that used for deletion, but with different landing pad plasmids and donor plasmids. In the first step, *E*. *coli* DH1 cells were transformed with pCNA and pBDC-Yi. Then, the same operation was carried out as the first step of genomic deletion. To confirm potential recombinant colonies, PCR was carried out using the primers Y-0 and Y-1, and the correct product was about 2300 bp in length. LB agar plates containing Amp were used to verify the elimination of the helper plasmid, pCNA, in positive colonies. The second step was the transformation of the strain with pSNK and pBRIS-*GC*. Cells were plated on LB agar plates containing Kan and Tc and incubated for about 20 h at 30°C. A colony was picked and suspended in 5 mL LB medium containing Kan and Tc; the cells were then incubated overnight at 30°C with shaking. Samples were diluted in 5 mL LB medium supplemented with Kan, and l-arabinose was added when the OD_600nm_ reached 0.2–0.3 to induce the expression of λ-Red proteins and I-SceI endonuclease. Cells were then incubated at 30°C for 4 h, after which they were diluted 100× and grown overnight on LB agar plates at 42°C. Cm-sensitive colonies were confirmed by PCR using the primers Y-0 and Y-1, and positive colonies yielded a product of about 4500 bp. The donor plasmid pBRIS-*GC* was cured by I-SceI endonuclease. To confirm the elimination of plasmids pSNK and pBRIS-*GC*, positive colonies were plated on LB agar plates containing Kan and LB agar plates containing Tc, respectively.

## Results

### Strategy of markerless deletion

**Fig 3:**
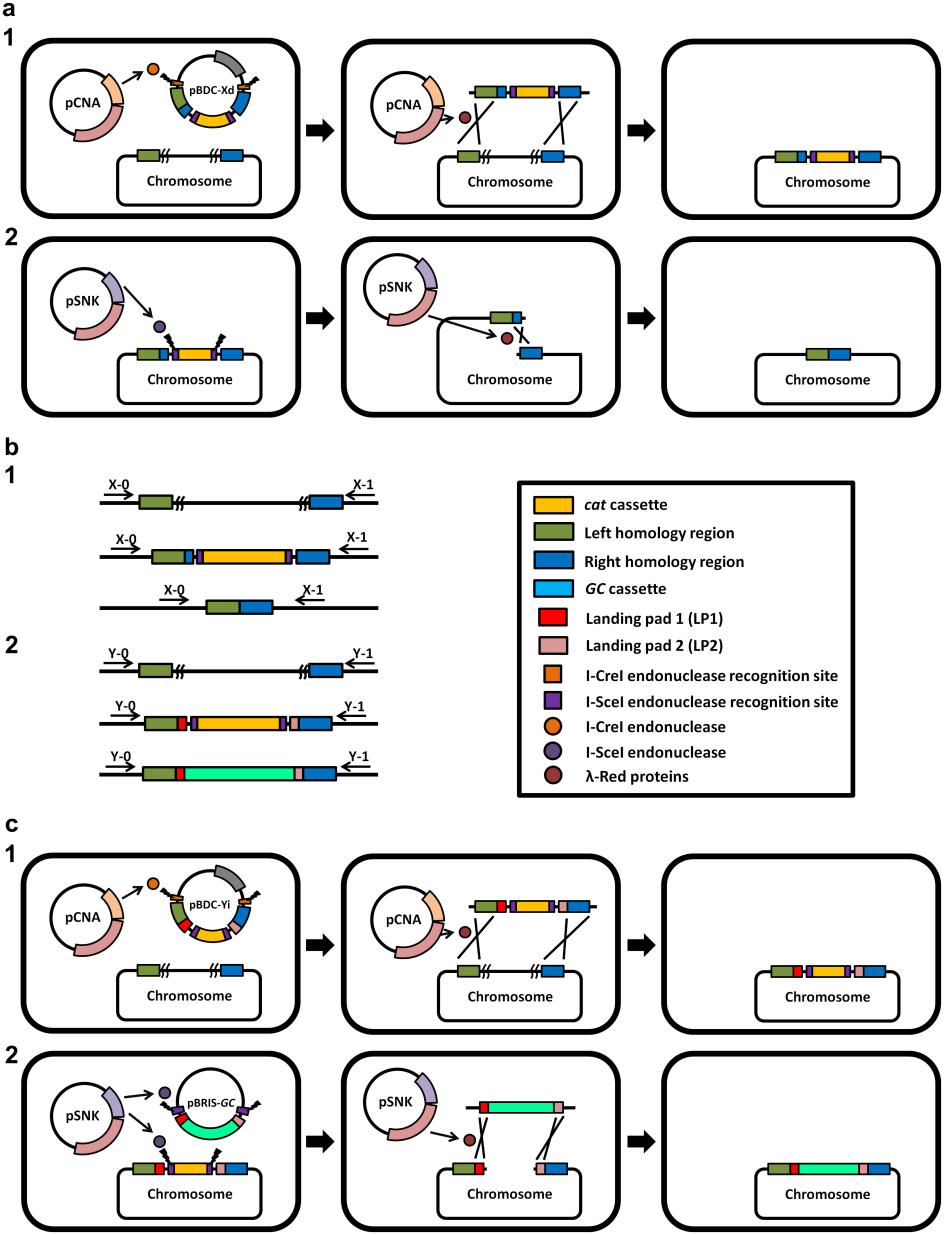
Strategy for markerless deletion and genomic replacement. (a) Progress of markerless deletion. In the first step, the pCNA and pBDC-Xd plasmids were transformed into *E*. *coli* DH1. With the addition of l-arabinose, pCNA expressed I-CreI endonuclease and λ-Red recombinases. The plasmid pBDC-Xd was digested by I-CreI endonuclease to release the landing pad fragment, which was the substrate for recombination. The landing pad fragment integrated into the *E*. *coli* chromosome. In the second step, the helper plasmid pSNK providing I-SceI endonuclease function was transformed into the host. Finally, the chromosome was cleaved by I-SceI endonuclease at the integration site. Recombination with the two homology regions, 50 bp of the right side of the left homology arm and left side of the right homology arm, led to chromosome repair with DSB-mediated recombination and resulted in a clean deletion. (b) Events of identifying markerless deletion and genomic replacement. In the protocol for markerless deletion, samples were verified by PCR with the primers X-0/X-1 (X = 1, 2, 5, 7, 8, 19, 55, or 63). Positive colonies were about 2300 bp in the first step and about 1000 bp in the second step. In the protocol for genomic replacement, samples were verified by PCR with the primers Y-0/Y-1 (Y = 8, 23, 57, or 64). Positive colonies were about 2300 bp in the first step and about 4500 bp in the second step. (c) Genomic replacement protocol. pBDC-Yi was employed as a landing pad plasmid in the first step. In the second step, the host cells were transformed with pSNK and pBRIS-*GC* (donor plasmid). pSNK expressed I-SceI endonuclease and λ-Red recombinases when l-arabinose was added. The donor plasmid and chromosome were cleaved at I-SceI endonuclease recognition sites. The integration of the donor fragment was accomplished by the expression of the λ-Red system.

Lee et al reported the method of “Gene Doctoring,” which involves two steps, i.e., inserting and delivering the Kan-resistance gene [13]. The I-SceI endonuclease was used for generating the target DNA fragment *in vivo* in the first step, and the FLP recombinase was used for breaking the chromosome in the second step, leaving a FLP scar [13]. In this study, I-SceI endonuclease was employed in the second step to achieve markerless deletion, and another endonuclease I-CreI was employed in the first step. First, the helper plasmid pCNA and the landing pad plasmid pBDC-Xd were transformed into *E*. *coli* DH1 cells. Second, transformation to recombinant cells was achieved by insertion of the plasmid pSNK. The helper plasmid pCNA contained the genes of λ-Red recombinases and I-CreI endonuclease under the control of *P*_*ara*_ (l-arabinose-inducible promoter; Fig 1). There were two I-CreI recognition sites and a *sacB* cassette in the backbone of pBDC-Xd. Between the I-CreI recognition sites, there were two 500-bp homologous arms (XL and XR) and a *cat* cassette flanked by two I-SceI recognition sites. With the help of the λ-Red system and I-CreI, the homologous fragment generated by double-strand breaks (DSBs) was integrated into the genome to replace the nonessential region (Fig 3a-1 and 3b-1).

Subsequently, the recombinant was transformed with pSNK (Fig 1) carrying the genes of Red recombinases and I-SceI endonuclease. After inducing the enzymes, the *cat* cassette was excised by I-SceI, and the generated DSB was repaired through Red recombination by a 50-bp homologous sequence in the XL arm (Fig 3a-2 and 3b-1).

### Strategy of genomic replacement

The genomic replacement strategy was similar to the deletion strategy. In the first step, the landing pad plasmid pBDC-Yi included a modified *cat* cassette, which was flanked by I-CreI sites, landing pads (LP1 and LP2), and homologous arms (YL and YR), that was transformed into *E*. *coli* DH1 with the helper plasmid pCNA. After induction with l-arabinose, the *cat* cassette was integrated into the genome to replace the nonessential region (Fig 3c-1). Subsequently, the recombinant was transformed with the helper plasmid pSNK and donor plasmid pBRIS-*GC*. Upon the action of I-SceI endonuclease and Red recombinases, the chromosome DSB was produced by excision of the *cat* gene and then repaired with the lycopene expression flux (designated the *GC* cassette) flanked by LP1 and LP2, which was released from pBRIS-*GC* (Fig 3b-2 and 3c-2). For example, lycopene expression flux was employed for genomic replacement because the native *E*. *coli* could not produce lycopene, which can be easily detected on a quantitative basis. Compared with that in the genomic replacement method of Kuhlman and Cox [14], in this study, the target DNA fragment was generated *in vivo* with the assistance of the I-CreI endonuclease, without the need of electroporation.

### Markerless deletion of nonessential regions in the chromosome

**Fig 4:**
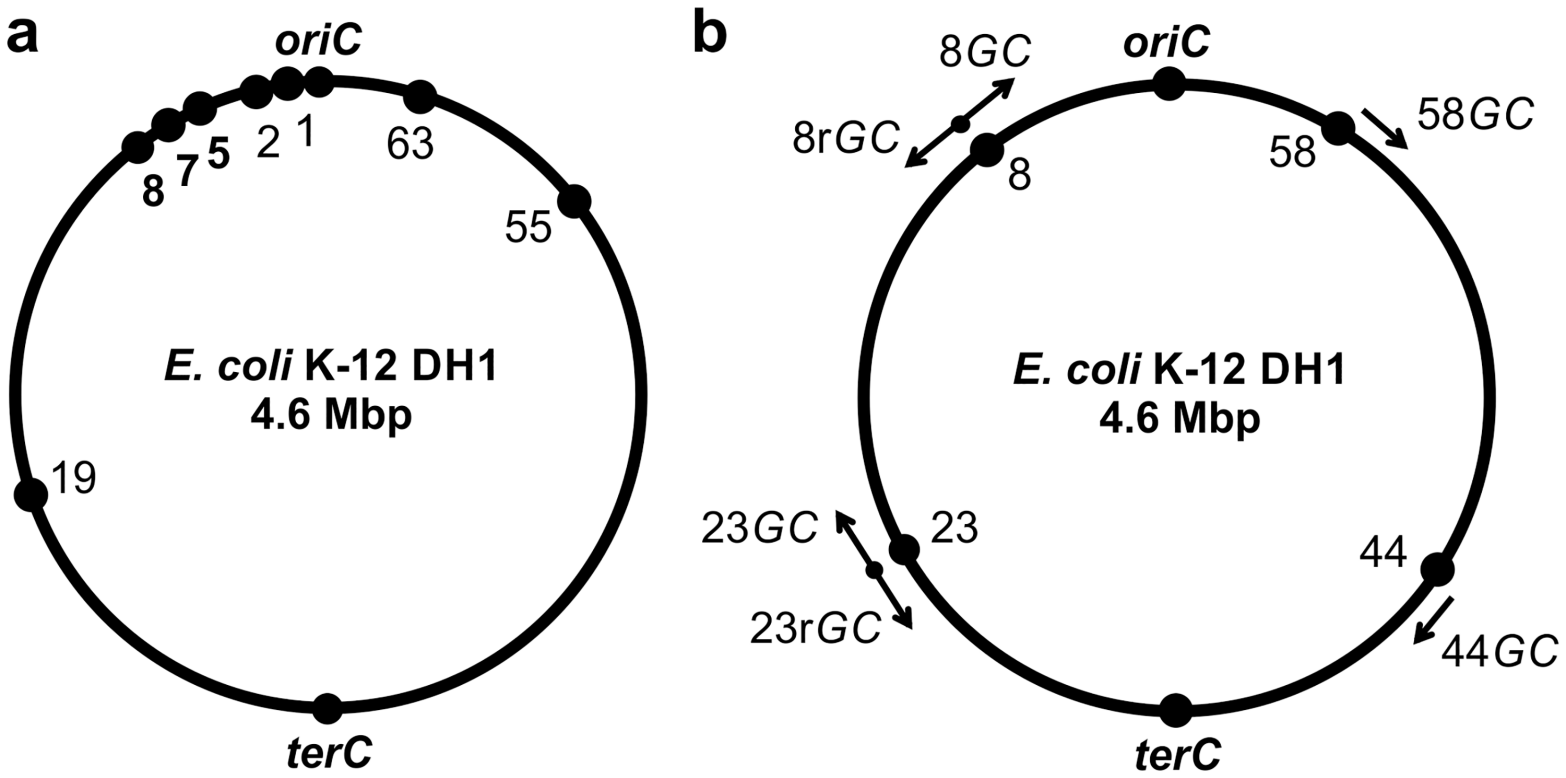
Chromosomal modification positions. (a) Eight nonessential regions were chosen for markerless deletion. (b) *GC* flux was integrated into four positions, symmetrically distributed throughout the genome. The arrows denote the direction of gene expression.

Sixty-four nonessential regions of *E*. *coli* DH1 (S4 Table) were obtained by our laboratory through sequence alignment, using Mauve software, between the genome of *E*. *coli* DH1, miniMG1655, and miniW3110 [17,18]. Eight nonessential regions (Fig 4a and Table 1) in the *E*. *coli* DH1 chromosome were selected (ranging from 2.4 to 105 kb in length). All eight regions were successfully deleted individually using this method, generating DH-1d, DH-2d, DH-5d, DH-7d, DH-8d, DH-19d, DH-55d, and DH-63d, respectively. The deletion region was sequenced using primer X-0, and the DNA sequencing results are shown in S4 Fig.

**Table 1:** Efficiency of pCNA/pSNK mediated deletions.

Target genomic region		First replacement		Selection efficiency of second step
		Selection efficiency^a^	Recombination efficiency^b^	
First	9.9 kb / 6254–16165	1/19 (5.3%)	2/20 (10%)	15/19 (78.9%)
Second	11.1 kb / 17432–28605	2/4 (50%)	1/40 (2.5%)^c^	17/18 (94.4%)
Fifth	2.4 kb / 230444–232865	9/9 (100%)	9/20 (45%)	7/17 (41.2%)
Seventh	3.9 kb / 299113–303017	1/5 (20%)	4/20 (20%)	7/15 (46.7%)
Eighth	16.4 kb / 413909–430258	1/9 (11.1%)	2/20 (10%)	6/19 (31.6%)
19^th^	8.3 kb / 1359035–1367343	8/19 (42.1%)	3/20 (15%)	17/19 (89.5%)
55^th^	104.4 kb / 3912835–4017200	16/18 (88.9%)	3/20 (15%)	15/17 (88.2%)
63^th^	19.8 kb / 4434265–4454029	4/19 (21.1%)	1/20 (5%)	6/14 (42.9%)
23^rd^	9.2 kb / 1487032–1496241	—	—	—
44^th^	15.5 kb / 3033115–3048652	—	—	—
58^th^	16.4 kb / 4184313–4200717	—	—	—

^a^The selection efficiency was calculated as the number of positive colonies relative to the total number of colonies verified by PCR.

^b^The recombination efficiency was the number of successful integration events per total number of cells.

^c^Among the first twenty colonies, none was positive and thus, another twenty colonies were picked and verified by PCR.

The selection efficiency of the Cm-resistance cassette for entry from the *E*. *coli* DH1 chromosome was 5.3–100% (Table 1). Additionally, the findings showed that there were no significant correlations between the size of the deletion region and the selection efficiency of deletion. For example, the efficiency was only 5.3% of the first nonessential region (9.9 kb) in the first step of genomic deletion. The size and location of the second nonessential region (11.1 kb) were similar to those of the first nonessential region; however, a much higher selection efficiency (50%) was achieved. The sizes of the fifth region and 55^th^ region were 2.4 and 104.4 kb, respectively, with efficiencies of 100% and 88.9%, respectively (Table 1). To further investigate the recombination efficiency, serial dilutions of the same cultures were plated on LB agar plates containing Cm and LBS agar plates, respectively. Theoretically, six types of mutants could be achieved after addition of l-arabinose and culture for 4 h in the first step of genomic deletion (S5 Fig). Mutants 1, 3, 4, and 6 could grow on LBS agar plates, whereas mutants 1, 2, 3, 5, and 6 could grow on LB agar plates containing Cm. The OD_600nm_ of undiluted cultures was 2.0–2.6; however, only 6–36 colonies were obtained on LBS plates at a dilution of 10×, and no colonies were observed at a dilution of 1000×. This result indicated that there were extremely low proportions of mutants 1, 3, 4, and 6 in cultures. Notably, 27–132 colonies were obtained on LB plates containing Cm with a dilution of 10^6^×. Thus, most cells were either mutant 2 or mutant 5, and their amounts were expected to be 10^4^–10^6^ higher than those of mutants 1, 3, 4, and 6. Thus, recombination efficiency could be calculated as the number of mutant 2 relative to the total number of mutants 2 and 5, regardless of whether both mutants 2 and 5 had residual pBDC plasmids. Twenty colonies were picked from LB agar plates containing Cm and verified by PCR using primers X-0 and X-1. A relatively high recombination efficiency of 2.5–45% was achieved (Table 1). Additionally, the recombination efficiency was not correlated with either selection efficiency or the size of the targeted region (Table 1). The positive recombinants achieved in the second step depended on I-SceI cutting and DSB repair, with a selection efficiency of 31.6–94.4% (Table 1).

### Lycopene flux integration into the chromosome to efficiently replace nonessential regions

The *GC* cassette was successfully inserted into four loci of *E*. *coli* DH1, i.e., the 8^th^, 23^rd^, 58^th^, and 64^th^ nonessential regions, distributed symmetrically throughout the chromosome (Fig 4b), generating DH-8*GC*, DH-23*GC*, DH-58*GC*, and DH-64*GC*, respectively. In order to compare the lycopene expression level with the transcription direction of *GC* in the chromosome, we also integrated the *GC* cassette in a counter-clockwise direction to generate DH-8r*GC* and DH-23r*GC*.

### Expression of lycopene integrated into the chromosome in different loci and directions

The collected mutants that could biosynthesize lycopene are shown in S6 Fig, indicating that the lycopene production level varied with the position and direction of integration (Table 2 and Fig 4b). When the *GC* transcription direction was the same as the replication direction, a five-fold difference in lycopene production was observed for the different integrated loci (DH-8r*GC*, DH-23r*GC*, DH-44*GC*, and DH-58*GC*), and there were no obvious relationships between the expression level and the distance to the replication site [19,20]. The integrated gene expression may be influenced by the structure of DNA nearby the integration position and by its neighboring upper gene expression [19]. Further, the integration direction had a strong effect on lycopene production. There was a more than 10-fold difference in lycopene levels between DH-23r*GC* and DH-23*GC* (Table 2). This contributed to lycopene production when the lycopene flux transcription direction was the same as the replication direction (Table 2 and Fig 4b). These results manifested that the expression direction of a gene strongly affected its expression to a greater degree than the chromosomal location. This conclusion is expected to guide further research of gene regulation and metabolic optimization. Interestingly, the highest expression strain DH1-23r*GC* had the lowest growth ability, potentially due to the excess consumption of isopentenyl diphosphate (IPP), implying that increasing the supply of IPP will be useful for high-level lycopene production [21].

**Table 2:** Strains used for lycopene production.

Strain	DH-8*GC*	DH-23*GC*	DH-44*GC*	DH-58*GC*	DH-8r*GC*	DH-23r*GC*
Titer (mg/L)	0.52 ± 0.07	0.71 ± 0.18	1.85 ± 0.25	1.70 ± 0.11	3.63 ± 0.21	8.36 ± 0.27
OD_600nm_	28.85 ± 1.01	28.03 ± 1.04	27.88 ± 0.84	28.33 ± 0.44	25.72 ± 0.63	25.13 ± 0.88

## Discussion

Our method exploited the advantages of genomic deletion from studies by Yu et al, Lee et al, and Kuhlman et al [11,13,14] and has the added feature of genomic replacement and elimination of the ~80-bp DNA scar. Compared with the existing electroporation method [5–7, 11], our method does not require a high-voltage electroporation apparatus. In addition, our landing pad plasmids and donor plasmids could be re-used at any time and have long-term storage capacity, without the requirement for generating PCR target fragments before transformation.

To improve recombination, several units have been employed in the λ-Red system, including FLP recombinase, I-SceI endonuclease, and the *sacB* counter-selection marker [6, 11–14]. In this study, we employed I-CreI endonuclease to act on I-CreI endonuclease sites of the landing pad plasmid and generated target DNA fragments *in vivo* in the first step of genomic modification. The selection and recombination efficiencies were 5.3–100% and 2.5–45%, respectively. The selection efficiency was lower than that described by Yu et al (70–100%) [11]. The unwanted colonies were thought to be related to the inefficiency of *sacB* counter-selection—some Cm-resistant colonies formed due to having an intact plasmid, instead of chromosomal integration of the Cm-resistance cassette. However, high recombination efficiency was achieved, which was 10^4^–10^7^ higher than that of the electroporation method (3.5 × 10^−6^ [12] and 2.9 × 10^−8^ [13] respectively). The achieved high recombination efficiency could be explained by the observation that nearly all cells yielded target DNA fragments and that the 500-bp homology region was long enough for recombination. Thus, the low recombination efficiency of the electroporation method could be explained by the low efficiency of electroporation. After addition of l-arabinose and culture for 4 h, most cells had the residual landing pad plasmid; thus, *sacB* counter-selection was necessary for pBDC plasmids. This study expanded our understanding of available elements for genomic modification. To the best of our knowledge, this is the first time that the I-CreI endonuclease and *sacB* counter-selection marker have been combined as assistant units to eliminate the plasmid and generate target DNA fragments *in vivo* for genomic modification. Compared with the I-CreI endonuclease, I-SceI showed higher efficiency of incision and introduction of DSBs both at the landing pad region of the chromosome and in high-copy donor plasmids without the need for the *sacB* cassette [14], thereby increasing the recombination efficiency by 5000× [22]. To eliminate the pBDC plasmids completely, further studies are needed to examine the evolution of the I-CreI endonuclease or to identify another endonuclease with higher efficiency *in vivo*. Thus, a one-step λ-Red recombination system could be established based on this method because of its high recombination efficiency.

## Supporting information

S1 Fig Plasmid containing the λ-Red system or endonuclease gene used in this study. (DOCX)

S2 Fig Construction of helper plasmids pKOBEGK, pSNA, pSNK, and pCNA. (DOCX)

S3 Fig Construction of the donor plasmid pBRIS-*GC*. (DOCX)

S4 Fig Results of markerless deletion. The deletion region was confirmed by sequencing with primer X-0, here, X = 1, 2, 5, 7, 8, 19, 55, or 63.
(DOCX)

S5 Fig Six types of mutants theoretically obtained before being plated in the first step of markerless deletion. (DOCX)

S6 Fig Collected strains before extraction by acetone. The *E*. *coli* cultures (500 μL) were centrifuged at 11,340 × *g* for 3 min. The supernatant was discarded.
(DOCX)

S1 Table Strains and plasmids used in this study. (DOCX)

S2 Table Sequences of *I-SceI*, *I-CreI*, and *GC* cassette. (DOCX)

S3 Table Primers used in this study. (DOCX)

S4 Table Nonessential sequences in the *Escherichia coli* DH1 genome (Our laboratory). (DOCX)
