# Correction: An electroporation-free method based on Red recombineering for markerless deletion and genomic replacement in the *Escherichia coli* DH1 genome

**DOI:** 10.1371/journal.pone.0229072

**Published:** 2020-02-06

**Authors:** Yanlong Wei, Pingping Deng, Ali Mohsin, Yan Yang, Huayan Zhou, Meijin Guo, Hongqing Fang

The following information is missing from the Data Availability statement: The plasmids described in this study are available from Addgene:

plasmid pBDC, Addgene ID: 135188

plasmid pBDK, Addgene ID: 135190

plasmid pCNA, Addgene ID: 135191

plasmid pSNA, Addgene ID: 135192

plasmid pSNK, Addgene ID: 135193

There is an error in S2 Table. The fourteenth base I-SceI should be “T,” not “A.” The correct S2 Table provided here shows the whole sequence of plasmid pSNA (Addgene ID: 135192).

## Supporting information

S2 Table Sequences of *I-SceI*, *I-CreI*, and *GC* cassette. (DOCX)
