# Supplementary figures and images for: An electroporation-free method based on Red recombineering for markerless deletion and genomic replacement in the Escherichia coli DH1 genome
Source: PLoS One. 2017 Oct 24;12(10):e0186891. doi: 10.1371/journal.pone.0186891 (PMC5655456; doi:10.1371/journal.pone.0186891)

**
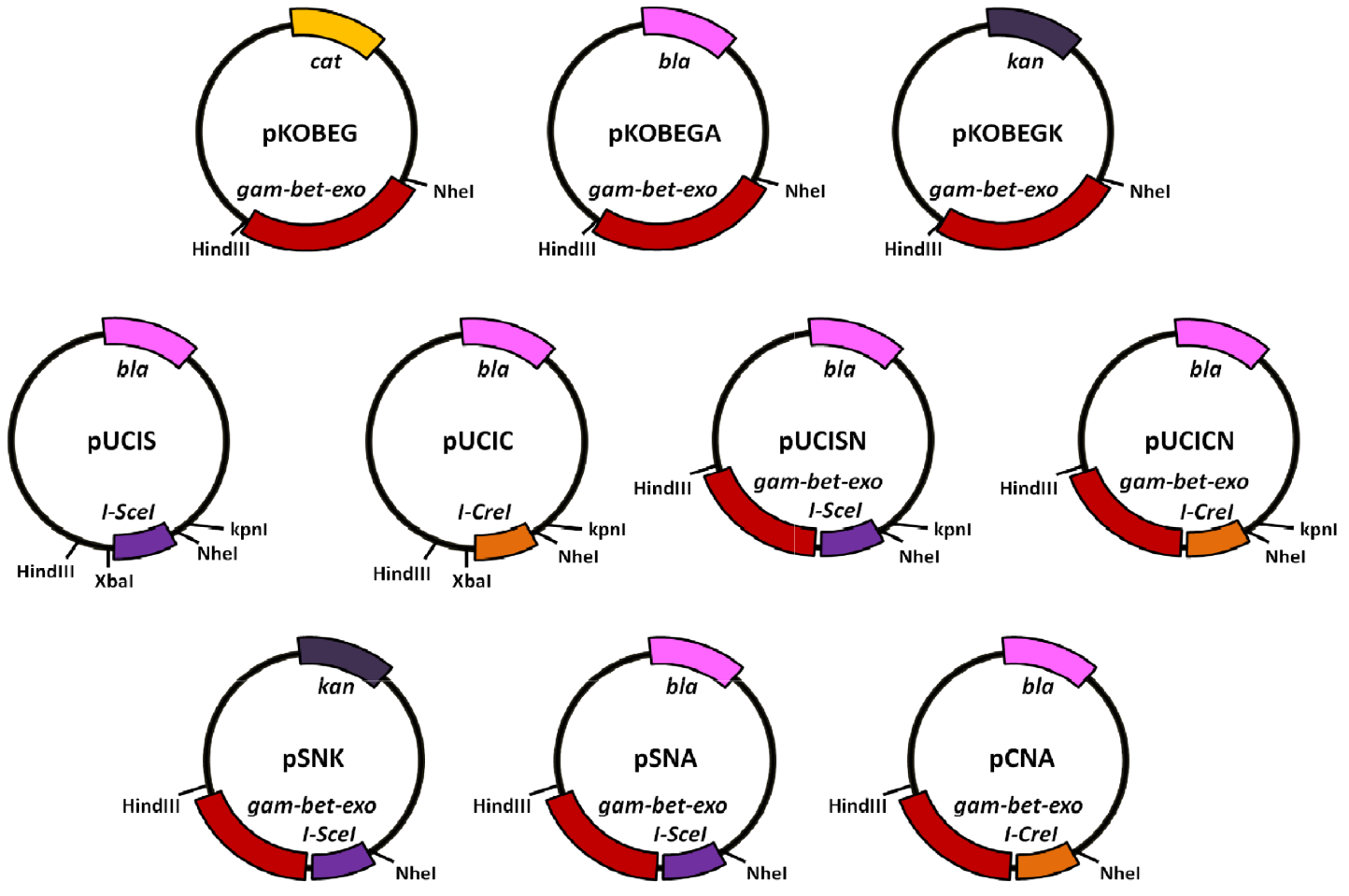
**

**S1 Fig. Plasmid containing the λ-Red system or endonuclease gene used in this study.**

Supplement: S1 Fig — (DOCX) [file pone.0186891.s001.docx]
