# Supplementary material for: An electroporation-free method based on Red recombineering for markerless deletion and genomic replacement in the Escherichia coli DH1 genome
Source: PLoS One. 2017 Oct 24;12(10):e0186891. doi: 10.1371/journal.pone.0186891 (PMC5655456; doi:10.1371/journal.pone.0186891)
Supplement: S2 Fig — (DOCX) [file pone.0186891.s002.docx]

**
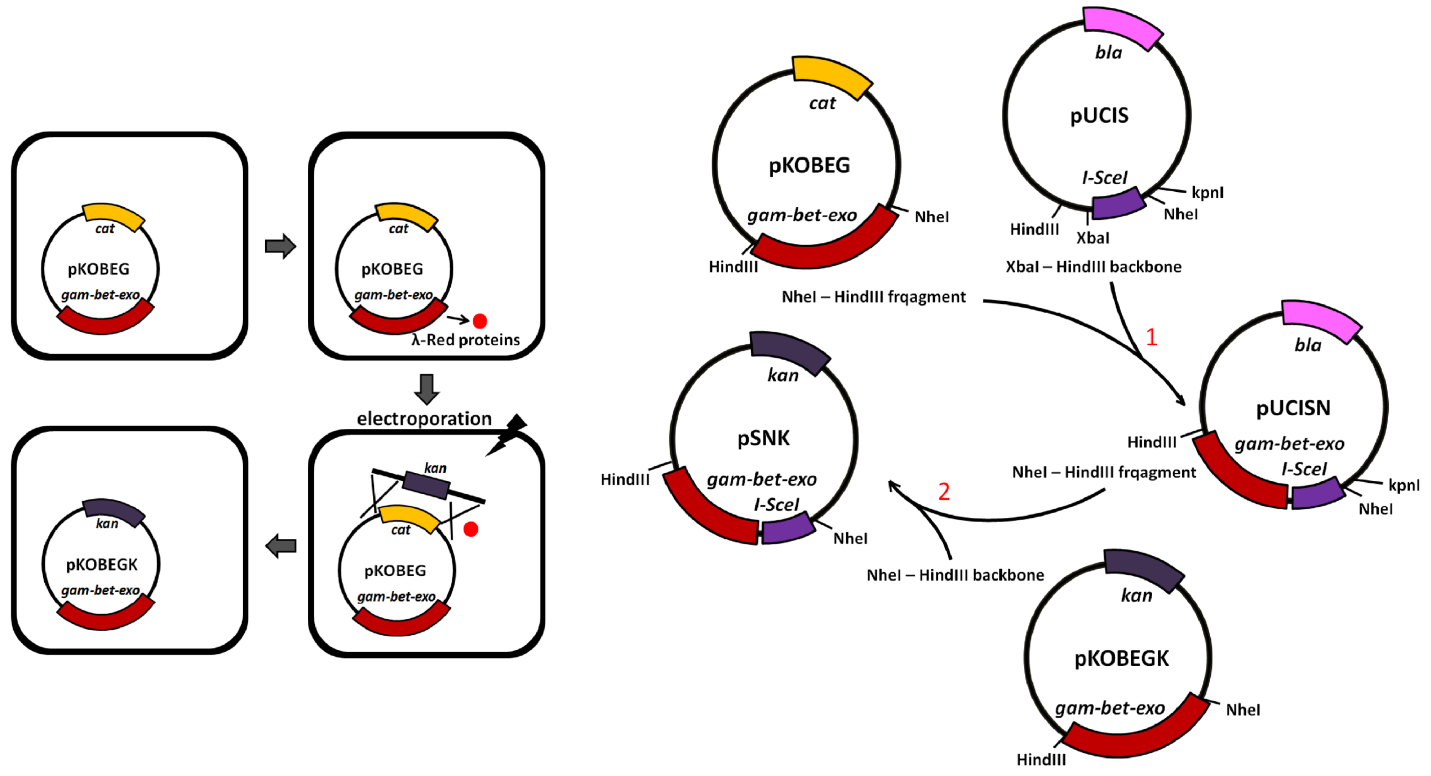
**

**S2 Fig.** **Construction of helper plasmids pKOBEGK, pSNA, pSNK, and pCNA.**

Plasmids pKOBEG and pKOBEGA were kindly provided by Christophe d'Enfert [5]. To create pKOBEGK, the target DNA fragment containing the Kan-resistance gene flanked by 50-bp homology sequences of the pKOBEG backbone was amplified by PCR from pET28a with primers KC5 and KC3, and the product was then incubated with *Dpn*I. DH5α cells containing pKOBEG were induced by l-arabinose to produce λ-Red proteins and then washed three times with water to create competent cells. The resulting target fragment was electroporated into competent cells (1870 V, ECM399; BTX, USA), and cells were then plated on Kan agar medium. Positive colonies can grow in Kan medium but cannot grow in Cm medium. Finally, the plasmid was sequenced by GENEWIZ Company using the backbone primer. The isolated λ-Red fragment (*gam-bet-exo* gene) was excised from pKOBEG using *Nhe*I and *Hind*III, followed by insertion into *Xba*I and *Hind*III sites in the vector pUCIS to create the plasmid pUCISN. The isolated *I-SceI-*λ-Red gene fragment from pUCISN was excised using *Nhe*I and *Hind*III, followed by insertion into the corresponding sites in the plasmid pKOBEGA and pKOBEGK to create pSNA and pSNK, respectively. The construction method for pCNA was the same as that for pSNA, with the vector changed from pUCIS to pUCIC.
