# Supplementary material for: An electroporation-free method based on Red recombineering for markerless deletion and genomic replacement in the Escherichia coli DH1 genome
Source: PLoS One. 2017 Oct 24;12(10):e0186891. doi: 10.1371/journal.pone.0186891 (PMC5655456; doi:10.1371/journal.pone.0186891)
Supplement: S3 Fig — (DOCX) [file pone.0186891.s003.docx]

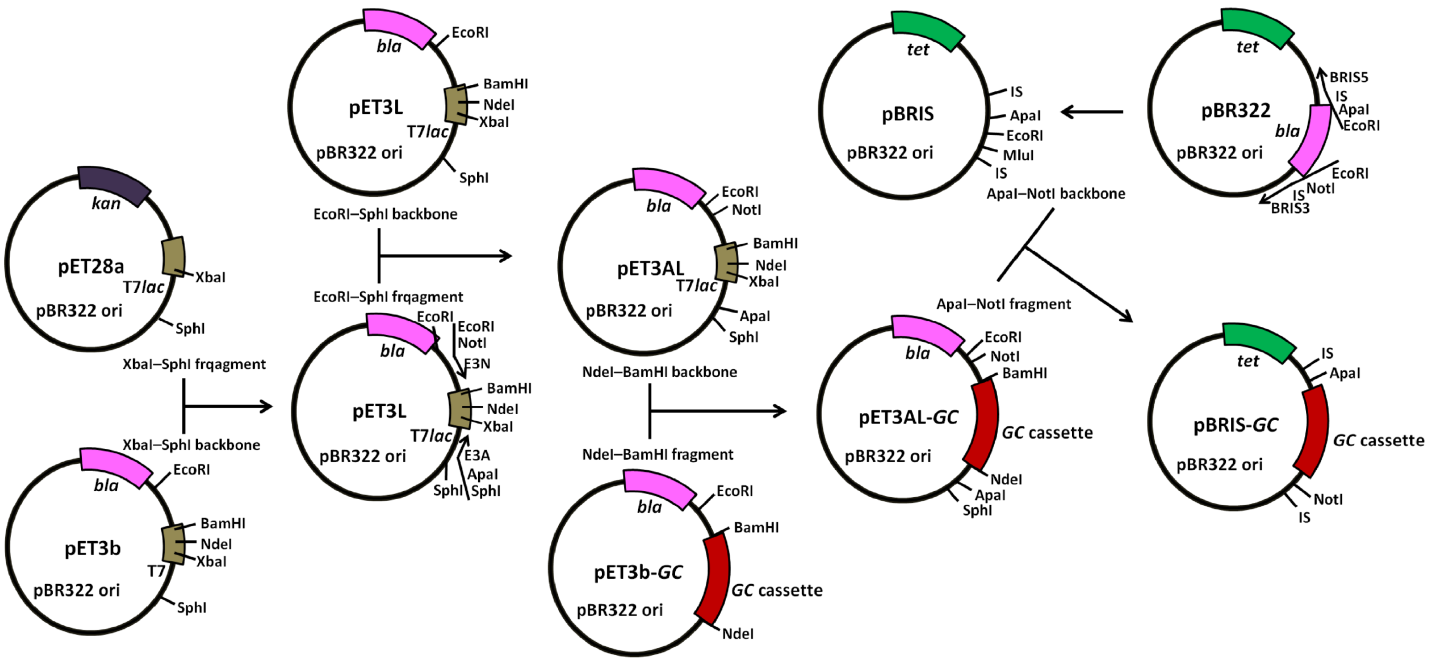


**S3 Fig.** **Construction of the donor plasmid pBRIS-*GC*.**

The *idsA* gene was optimized for expression in *E. coli*, artificially synthesized, and cloned into the *Nde*I/*BamH*I site of pUC57 to generate pUC-*idsA* by GENEWIZ Company. In pUC-*idsA*, between the termination codon of the *idsA* gene and the *BamH*I site, *Xho*I, *Mun*I, and *Not*I sites were added. The plasmid pUC-*idsA* was excised using *Nde*I and *BamH*I, followed by insertion into the corresponding sites in the vector pET3b to generate pET3b-*idsA*. The CIB fragment containing the *crtI* and *crtB* genes was amplified by PCR from the genome of *P. agglomerans* using the primers CRT4/CRT5. To construct the *GC* cassette, the *BamH*I and *Bgl*II sites of the CIB fragment needed to be eliminated. To eliminate the *BamH*I site of CIB fragment, the fusion PCR method was used. First, the CIB1a fragment (the 1.7-kb sequence before the *BamH*I site in the CIB fragment) was obtained by PCR from the CIB fragment using the primers CRT4 and CRT6. Then, the CIB1b fragment (the 0.7-kb sequence after the *BamH*I site in the CIB fragment) was obtained by PCR from the CIB fragment using the primers CRT7 and CRT5. Finally, the CIB1 fragment (gene cluster *crtI-crtB* with elimination of the *BamH*I site) was obtained by PCR using primers CRT4/CRT5 and the fragment CIB1a/CIB1b mixture as the template.

The *Bgl*II site of the CIB1 fragment was subsequently eliminated. First, the CIB2a fragment (the 0.7-kb sequence before the *Bgl*II site in the CIB1 fragment) was obtained by PCR from the CIB1 fragment using primers CRT4 and CRT8. Next, the CIB2b fragment (the 1.7-kb sequence after the *Bgl*II site in the CIB1 fragment) was obtained by PCR from the CIB1 fragment using primers CRT5 and CRT9. Then, the isolated CIB2a fragment was digested by *Xho*I and *BamH*I to generate CIB2a-XB, and the isolated CIB2b fragment was digested by *Bgl*II and *EcoR*I to generate CIB2b-BE. Finally, these two isolated fragments were together inserted into *Xho*I/*Mun*I sites of pET3b-*idsA* to generate pET3b-*GC*.

The plasmid pBRIS-*GC* was the plasmid pBRIS carrying the *GC* cassette. To construct pBRIS, the fragment BRIS containing pBM1 ori and the Tc resistance gene, flanked by I-SceI recognition sites was amplified by PCR using the primers BRIS5/BRIS3 and pBR322 as a template. Then, the fragment was excised using *EcoR*I, followed by self-ligation to generate plasmid pBRIS. To insert the *GC* cassette into the backbone of pBRIS, a series of plasmids, i.e., pET3L, pET3AL, and pET3AL-*GC*, were generated. The plasmid pET3L contained the backbone of pET3b and the T7*lac* promoter of pET28a. To construct pET3L, plasmid pET28a was digested by *Sph*I and *Xba*I to generate a 260-bp *Sph*I-*Xba*I fragment. Then, the *Sph*I-*Xba*I fragment was inserted into the corresponding sites in the plasmid pET3b to create pET3L. The plasmid pET3AL was based on pET3L, which was inserted into the *Apa*I and *Not*I sites. To construct pET3AL, a 500-bp fragment (SphI-ApaI-T7*lac*-NotI-EcoRI) was obtained by PCR using primers E3A and E3N and pET3L as a template. Next, the fragment was excised by *EcoR*I and *Sph*I and then inserted into the corresponding sites of pET3L to generate pET3AL. The isolated *GC* cassette was excised using *Nde*I and *BamH*I from pET3b-*GC*, followed by insertion into the corresponding sites in the plasmid pET3AL to create pET3AL-*GC*. The *Apa*I-*Not*I fragment containing the *GC* cassette was excised using *Apa*I and *Not*I from pET3AL-*GC*, followed by insertion into the corresponding sites in the plasmid pBRIS to create pBRIS-*GC*.
