# Supplementary material for: An electroporation-free method based on Red recombineering for markerless deletion and genomic replacement in the Escherichia coli DH1 genome
Source: PLoS One. 2017 Oct 24;12(10):e0186891. doi: 10.1371/journal.pone.0186891 (PMC5655456; doi:10.1371/journal.pone.0186891)
Supplement: S4 Fig — The deletion region was confirmed by sequencing with primer X-0, here, X = 1, 2, 5, 7, 8, 19, 55, or 63. (DOCX) [file pone.0186891.s004.docx]

**
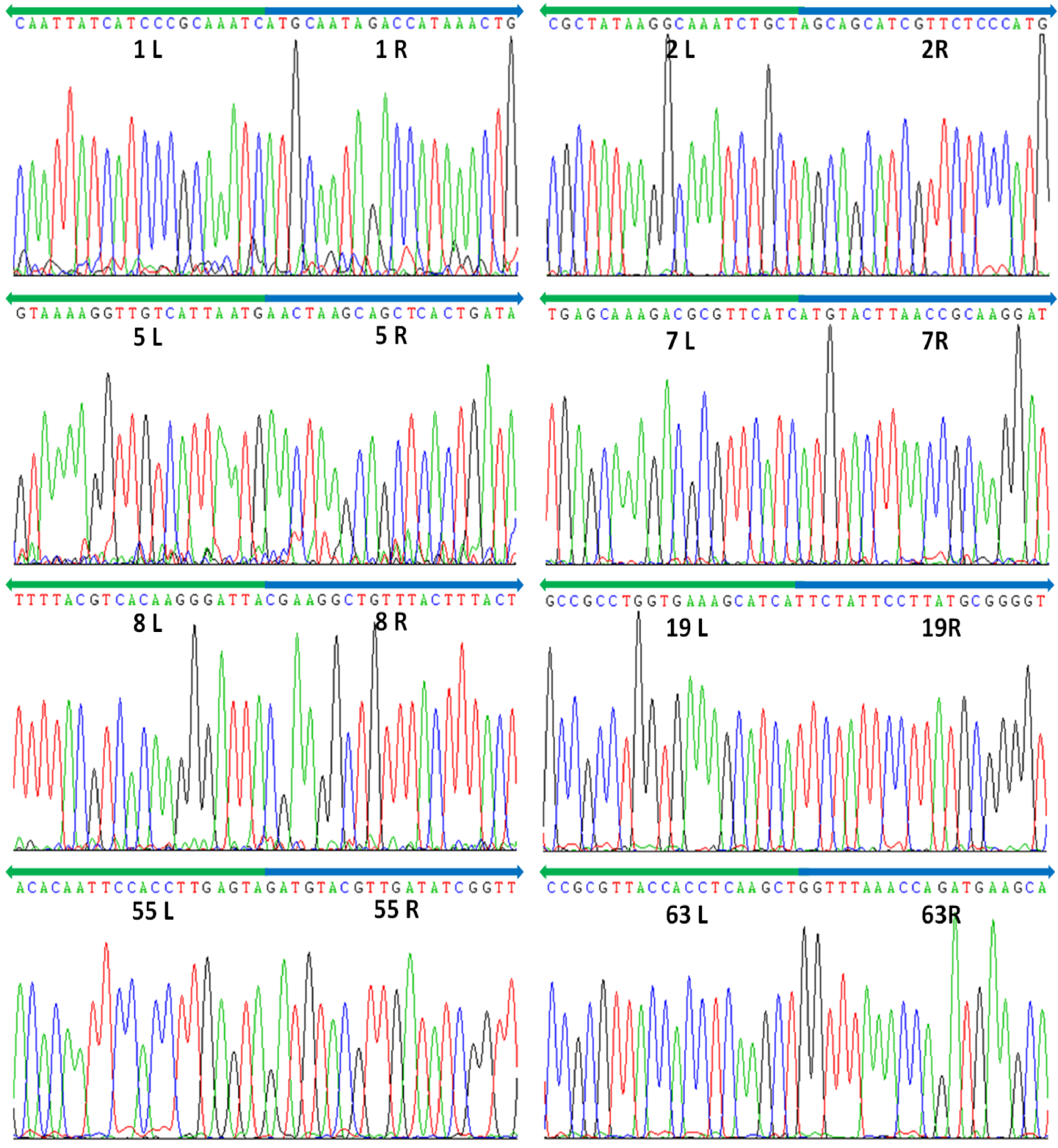
**

**S4 Fig. Results of markerless deletion.** The deletion region was confirmed by sequencing with primer X-0, here, X = 1, 2, 5, 7, 8, 19, 55, or 63.
