# Supplementary material for: An electroporation-free method based on Red recombineering for markerless deletion and genomic replacement in the Escherichia coli DH1 genome
Source: PLoS One. 2017 Oct 24;12(10):e0186891. doi: 10.1371/journal.pone.0186891 (PMC5655456; doi:10.1371/journal.pone.0186891)
Supplement: S5 Fig — (DOCX) [file pone.0186891.s005.docx]

**
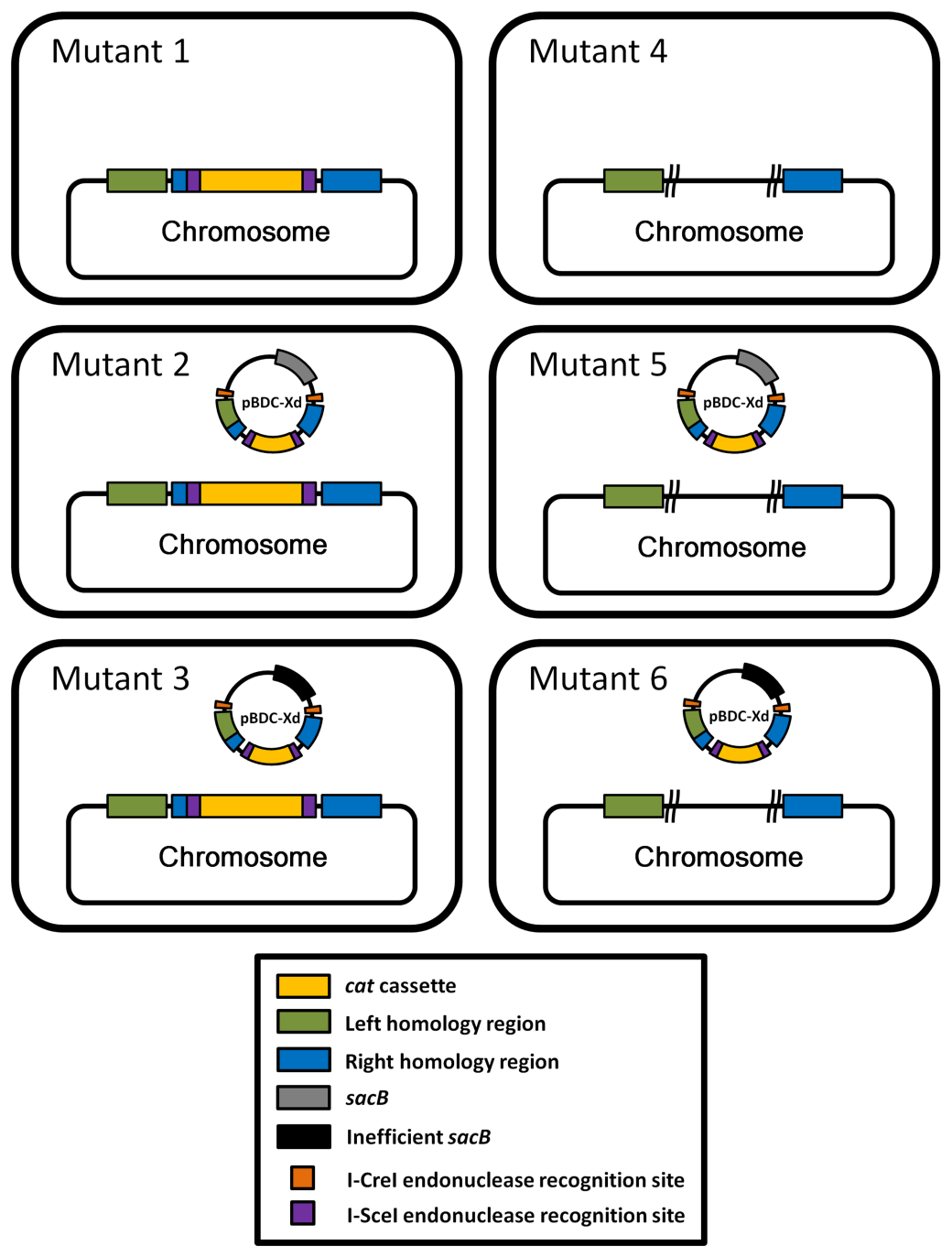
**

**S5 Fig.** **Six types of mutants theoretically obtained before being plated in the first step of markerless deletion.** Mutant 1 was a positive recombinant. Mutant 2 and 3 were positive recombinants with residual pBDC plasmids and pBDC carrying inefficient *sacB*, respectively. Mutant 4 was a negative recombinant. Mutant 5 and 6 were the mutant 4 with residual pBDC plasmids and pBDC carrying inefficient *sacB*, respectively.
