# Supplementary material for: An electroporation-free method based on Red recombineering for markerless deletion and genomic replacement in the Escherichia coli DH1 genome
Source: PLoS One. 2017 Oct 24;12(10):e0186891. doi: 10.1371/journal.pone.0186891 (PMC5655456; doi:10.1371/journal.pone.0186891)
Supplement: S6 Fig — The E. coli cultures (500 μL) were centrifuged at 11,340 × g for 3 min. The supernatant was discarded. (DOCX) [file pone.0186891.s006.docx]

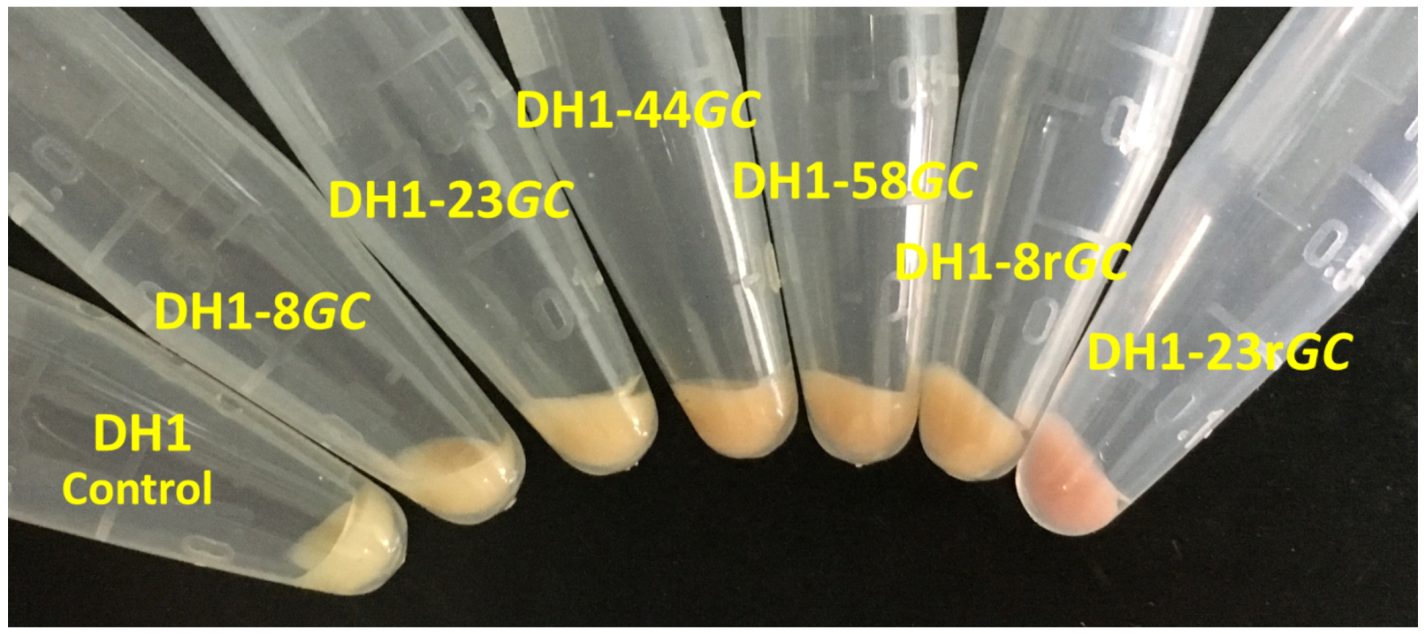


**S6 Fig.** **Collected strains before extraction by acetone.** The *E. coli* cultures (500 μL) were centrifuged at 11,340 × *g* for 3 min. The supernatant was discarded.
