# Supplementary material for: An electroporation-free method based on Red recombineering for markerless deletion and genomic replacement in the Escherichia coli DH1 genome
Source: PLoS One. 2017 Oct 24;12(10):e0186891. doi: 10.1371/journal.pone.0186891 (PMC5655456; doi:10.1371/journal.pone.0186891)
Supplement: S1 Table — (DOCX) [file pone.0186891.s007.docx]

**S1 Table. Strains and plasmids used in this study.**

| Name | Description | Reference |
| --- | --- | --- |
| Strains |  |  |
| *E. coli* DH5α | *deoR*, *recA*1, *endA*1, *hsdR*17(r_k_^-^ m_k_^+^), *phoA*, *supE*44, λ^-^, *thi*-1, *gyrA*96, *relA*1 | Our laboratory |
| *E. coli* DH1 | *supE*44, *hsdR*17, *recA*1, *endA*1, *gyrA*96, *thi*-1, *relA*1 | Our laboratory |
| DH-1d | *E. coli* DH1 Δ1^st^ nonessential region | This study |
| DH-2d | *E. coli* DH1 Δ2^nd^ nonessential region | This study |
| DH-5d | *E. coli* DH1 Δ5^th^ nonessential region | This study |
| DH-7d | *E. coli* DH1 Δ7^th^ nonessential region | This study |
| DH-8d | *E. coli* DH1 Δ8^th^ nonessential region | This study |
| DH-19d | *E. coli* DH1 Δ19^th^ nonessential region | This study |
| DH-55d | *E. coli* DH1 Δ55^th^ nonessential region | This study |
| DH-63d | *E. coli* DH1 Δ63^th^ nonessential region | This study |
| DH-8*GC* | *E. coli* DH1 Δ8^th^ :: *gps-crtIB* | This study |
| DH-23*GC* | *E. coli* DH1 Δ23^th^ :: *gps-crtIB* | This study |
| DH-44*GC* | *E. coli* DH1 Δ44^th^ :: *gps-crtIB* | This study |
| DH-58*GC* | *E. coli* DH1 Δ58^th^ :: *gps-crtIB* | This study |
| DH-8r*GC* | *E. coli* DH1 Δ8^th^ :: *gps-crtIB* (counter-clockwise) | This study |
| DH-23r*GC* | *E. coli* DH1 Δ23^th^ :: *gps-crtIB* (counter-clockwise) | This study |
| Plasmids |  |  |
| pKOBEG | repA101ts & oriR101, araC pBAD-*gam-bet-exo*, Cm^R^ | 5 |
| pKOBEGA | repA101ts & oriR101, araC pBAD-*gam-bet-exo*, Amp^R^ | 5 |
| pKOBEGK | repA101ts & oriR101, araC pBAD-*gam-bet-exo*, Kan^R^ | Our laboratory |
| pUC19 | pMB1 ori, *lacZα*, Amp^R^ | GENEWIZ Company |
| pUC57 | pMB1 ori, *lacZα*, Amp^R^ | GENEWIZ Company |
| pBR322 | pMB1 ori, Tc^R^, Amp^R^ | New England Biolabs, Inc. |
| pET3b | pBR322 ori, T7 promoter, Amp^R^ | EMD Biosciences (Novagen) |
| pET28a | pBR322 ori, f1 ori, T7*lac* promoter, *lacI*, Kan^R^ | EMD Biosciences (Novagen) |
| pACYCDuet-1 | p15A origin, T7*lac* promoter, *lacI*, Cm^R^ | EMD Biosciences (Novagen) |
| pUCIS | pUC19 derived, carrying *I-SceI* endonuclease gene, Amp^R^ | GENEWIZ Company |
| pUCISN | pUC19 derived, carrying *I-SceI* endonuclease gene-*gam-bet-exo*, Amp^R^ | Our laboratory |
| pUCIC | pUC19 derived,carrying *I-CreI* endonuclease gene, Amp^R^ | GENEWIZ Company |
| pUCICN | pUC19 derived, carrying *I-CreI* endonuclease gene-*gam-bet-exo*, Amp^R^ | Our laboratory |
| pCNA | pKOBEG derived, carrying *I-CreI* endonuclease gene at Nhe I site, Amp^R^ | Our laboratory |
| pSNA | pKOBEG derived, carrying *I-SceI* endonuclease gene at Nhe I site, Amp^R^ | Our laboratory |
| pSNK | pKOBEG derived, carrying *I-SceI* endonuclease gene at Nhe I site, Kan^R^ | Our laboratory |
| pBDC | p15A origin, *sacB* cassette, Cm^R^ | Our laboratory |
| pBDK | p15A origin, *sacB* cassette, Kan^R^ | Our laboratory |
| pBDC-1d | pBDC derived, carrying first homologous region | This study |
| pBDC-2d | pBDC derived, carrying second homologous region | This study |
| pBDC-5d | pBDC derived, carrying fifth homologous region | This study |
| pBDC-7d | pBDC derived, carrying seventh homologous region | This study |
| pBDC-8d | pBDC derived, carrying eighth homologous region | This study |
| pBDC-19d | pBDC derived, carrying 19^th^ homologous region | This study |
| pBDC-55d | pBDC derived, carrying 55^th^ homologous region | This study |
| pBDC-63d | pBDC derived, carrying 63^th^ homologous region | This study |
| pBDC-8i | pBDC derived, carrying eighth homologous region, LP1 and LP2 | This study |
| pBDC-23i | pBDC derived, carrying 23^rd^ homologous region, LP1 and LP2 | This study |
| pBDC-44i | pBDC derived, carrying 44^th^ homologous region, LP1 and LP2 | This study |
| pBDC-58i | pBDC derived, carrying 58^th^ homologous region, LP1 and LP2 | This study |
| pBDC-8ri | pBDC derived, carrying eighth homologous region, LP2 RCS and LP1 RCS | This study |
| pBDC-23ri | pBDC derived, carrying 23^rd^ homologous region, LP2 RCS and LP1 RCS | This study |
| pUC-*idsA* | pUC19 derived, carrying *A. fulgidus idsA*, Amp^R^ | GENEWIZ Company |
| pET3b-*idsA* | pET3b derived, carrying *A. fulgidus idsA*, Amp^R^ |  |
| pET3b-*GC* | pET3b derived, carrying *A. fulgidus idsA* and *P. agglomerans crtIB*, Amp^R^ | This study |
| pET3L | pET3b derived, T7*lac* promoter, Amp^R^ | This study |
| pET3AL | pET3L derived, carrying ApaI and NotI restriction site, Amp^R^ | This study |
| pET3AL-*GC* | pET3AL derived, carrying *A. fulgidus idsA* and *P. agglomerans crtIB*, Amp^R^ | This study |
| pBRIS | pBR322 derived, carrying I-SceI recognition site, Tc^R^ | This study |
| pBRIS-*GC* | pBRIS derived, carrying *A. fulgidus idsA* and *P. agglomerans crtIB*, Tc^R^ | This study |
