# Supplementary material for: An electroporation-free method based on Red recombineering for markerless deletion and genomic replacement in the Escherichia coli DH1 genome
Source: PLoS One. 2017 Oct 24;12(10):e0186891. doi: 10.1371/journal.pone.0186891 (PMC5655456; doi:10.1371/journal.pone.0186891)
Supplement: S2 Table — (DOCX) [file pone.0186891.s008.docx]

**S2 Table. Sequences of *I-SceI*, *I-CreI*, and *GC* cassette.**

|  | DNA sequence |
| --- | --- |
| *I-SceI* | GGTACCGCTAGCAGGAGGGTACCTATATGCATATGAAAAACATCAAAAAAAACCAGGTAATGAACCTCGGTCCGAACTCTAAACTGCTGAAAGAATACAAATCCCAGCTGATCGAACTGAACATCGAACAGTTCGAAGCAGGTATCGGTCTGATCCTGGGTGATGCTTACATCCGTTCTCGTGATGAAGGTAAAACCTACTGTATGCAGTTCGAGTGGAAAAACAAAGCATACATGGACCACGTATGTCTGCTGTACGATCAGTGGGTACTGTCCCCGCCGCACAAAAAAGAACGTGTTAACCACCTGGGTAACCTGGTAATCACCTGGGGCGCCCAGACTTTCAAACACCAAGCATTCAACAAACTGGCTAACCTGTTCATCGTTAACAACAAAAAAACCATCCCGAACAACCTGGTTGAAAACTACCTGACCCCGATGTCTCTGGCATACTGGTTCATGGATGATGGTGGTAAATGGGATTACAACAAAAACTCTACCAACAAATCGATCGTACTGAACACCCAGTCTTTCACTTTCGAAGAAGTAGAATACCTGGTTAAGGGTCTGCGTAACAAATTCCAACTGAACTGTTACGTAAAAATCAACAAAAACAAACCGATCATCTACATCGATTCTATGTCTTACCTGATCTTCTACAACCTGATCAAACCGTACCTGATCCCGCAGATGATGTACAAACTGCCGAACACTATCTCCTCCGAAACTTTCCTGAAATAATCTAGA |
| *I-CreI* | GGTACCGCTAGCTAAGAAGGAGATATACATATGGCTAATACCAAATATAACAAAGAGTTCCTGCTGTACCTGGCCGGCTTTGTGGACGGTGACGGTAGCATCATCGCTCAGATTAAACCAAACCAGTCTTATAAGTTTAAACATCAGCTGAGCTTGACCTTTCAGGTGACTCAAAAGACCCAGCGCCGTTGGTTTCTGGACAAACTAGTGGATGAAATTGGCGTTGGTTACGTACGTGATCGCGGTTCCGTTTCCGATTACATCTTAAGCGAAATCAAGCCGCTGCACAACTTCCTGACTCAACTGCAGCCGTTTCTGAAACTGAAACAGAAACAGGCAAACCTGGTTCTGAAAATTATCGAACAGCTGCCGTCTGCAAAAGAATCCCCGGACAAATTCCTGGAAGTTTGTACCTGGGTGGATCAGATTGCAGCTCTGAACGATTCTAAGACTCGTAAAACCACTTCTGAAACCGTTCGTGCTGTGCTGGACAGCCTGAGCGAGAAGAAGAAATCCTCCCCGGCGGCCGACTAATCTAGA |
| *GC* cassette | CATATGGATAGATTTCAGCGTTTGATTGCCATGCTGAAGGAGGAAATTGCGAAACGTGCCGAAATTATCAACAAAGCCATTGAAGAGCTTCTGCCGGAACGTGAGCCGATTGGTCTCTACAAAGCCGCACGTCATCTGATCAAAGCAGGTGGCAAGCGTCTGCGTCCTGTAATCAGCCTCTTAGCAGTCGAAGCCCTTGGTAAAGACTACAGAAAGATTATCCCGGCTGCTGTCAGCATTGAAACAATCCACAACTTCACCCTCGTGCATGACGACATCATGGACCGTGACGAGATGCGTCGTGGTGTTCCGACTGTACACAGAGTTTATGGTGAAGCGACTGCCATTTTAGCAGGCGACACACTCTTTGCTGAAGCCTTCAAGCTGCTGACAAAGTGCGATGTTGAGAGCGAGGGTATCAGAAAAGCTACAGAAATGCTTTCGGACGTTTGCATTAAAATTTGCGAGGGTCAGTACTACGACATGAGCTTTGAGAAAAAGGAGAGCGTTTCCGAGGAGGAGTATCTCAGAATGGTCGAGCTGAAGACCGGTGTGCTGATTGCAGCTTCTGCAGCATTACCTGCGGTGCTTTTTGGTGAGAGCGAGGAAATTGTAAAGGCGCTGTGGGACTACGGTGTTCTTAGCGGTATTGGCTTCCAGATCCAGGACGACCTGCTTGACCTGACTGAGGAGACCGGTAAGGACTGGGGTAGCGACCTGCTTAAAGGTAAGAAAACCCTGATTGTCATTAAGGCGTTCGAAAAGGGTGTGAAGCTGAAGACATTTGGTAAGGAAAAGGCGGACGTCTCTGAGATTAGAGATGATATCGAAAAGTTAAGAGAGTGTGGTGCGATTGATTACGCTGCCAGCATGGCAAGAAAGATGGCTGAAGAGGCGAAAAGAAAGCTCGAAGTTCTGCCTGAAAGCAAAGCCAAGGAAACACTGCTGGAACTTACCGACTTCTTGGTTACAAGAAAAAAGTAACTCGAGTAAGGAGGATATTTAGATGAATAGAACTACAGTAATTGGCGCAGGCTTTGGTGGTCTGGCTCTGGCCATTCGCCTTCAGGCGTCAGGCGTTCCCACCCGACTGCTGGAGCAGCGTGACAAGCCGGGCGGCCGGGCTTATGTCTATCAGGATCAGGGCTTCACGTTTGATGCCGGCCCCACGGTAATCACCGATCCCAGCGCCATTGAAGAGCTGTTCACTCTGGCGGGTAAAAAGCTCTCTGACTATGTCGAGCTGATGCCGGTGAAGCCGTTTTATCGCCTCTGCTGGGAGTCCGGCAAGGTGTTCAGTTATGACAACGATCAGCCCGCGCTGGAAGCGCAGATTGCCGCATTTAATCCGCGTGACGTTGAAGGATATCGGCGCTTTCTGGCCTATTCCCGAGCGGTGTTTGCTGAAGGCTATCTGAAGCTTGGCACCGTGCCGTTTCTGTCATTCCGCGACATGCTGCGGGCCGCGCCTCAGCTGGCAAAACTTCAGGCATGGCGCAGCGTTTACAGCAAAGTGGCGAGCTACATTGAAGATGAGCATCTGCGTCAGGCCTTCTCTTTCCACTCACTGCTGGTGGGCGGAAATCCGTTTGCCACTTCCTCAATCTATACCCTGATTCATGCGCTGGAACGTGAATGGGGCGTCTGGTTCCCGCGCGGTGGCACGGGCGCGCTGGTGCAGGGCATGGTGAAACTGTTTGAGGATCTGGGCGGCGAAGTGGAGCTCAATGCCAGCGTTGCCCGGCTGGAGACCCAGGAAAACAGGATTACCGCGGTGCACCTGAAAGATGGCCGGGTCTTCCCGACCCGCGCGGTTGCCTCCAACGCAGATGTGGTTCACACCTACCGCGAACTGCTGAGCCAGCACCCCGCTTCGCAGGCGCAGGGACGGTCACTGCAGAACAAACGCATGAGTAACTCGCTGTTTGTGATCTATTTTGGCCTGAATCATCATCACGATCAGCTGGCGCACCACACGGTCTGCTTTGGTCCGCGCTATCGTGAGTTGATTGATGAAATCTTTAACAAAGATGGCCTGGCAGAGGACTTCTCGCTCTATCTGCACGCGCCCTGCGTGACCGATCCCTCACTGGCACCGGAAGGCTGCGGCAGCTACTACGTGCTGGCGCCGGTACCGCACCTCGGCACCGCTGATATCGACTGGGCCGTTGAAGGTCCGCGCCTGCGCGATCGCATTTTCGACTATCTGGAACAGCATTACATGCCGGGCCTGCGTAGCCAGTTGGTCACGCATCGCATCTTCACGCCGTTTGATTTCCGCGATGAGCTGAATGCGTATCAGGGCTCGGCCTTCTCAGTGGAGCCGATCCTGACGCAAAGCGCCTGGTTCCGGCCTCACAACCGCGATAAAAATATTAATAATCTCTATCTGGTCGGTGCTGGTACCCATCCTGGCGCGGGTATTCCAGGGGTGATTGGCTCGGCCAAGGCTACCGCAGGATTGATGCTGGAGGATCTGGCTTGAATAGTCCGTCACTGCTTGATCATGCCGTAGACACCATGGAGGTGGGATCGAAAAGCTTTGCCACCGCGTCAAAACTGTTTGATGCCAAAACCCGACGCAGCGTGCTGATGCTCTACGCCTGGTGCCGTCACTGTGATGATGTGATTGACGATCAGGTCCTGGGATTCAGCAACGATACGCCATCGCTGCAATCTGCCGAACAGCGCCTGGCGCAGCTGGAGATGAAAACGCGTCAGGCCTATGCCGGTTCCCAGATGCATGAGCCCGCCTTTGCGGCCTTTCAGGAGGTGGCAATGGCGCACGATATTCTGCCTGCTTACGCTTTTGATCATCTGGCGGGCTTTGCGATGGACGTGCATGAGACACGCTATCAGACGCTGGATGATACGCTGCGTTACTGTTACCACGTCGCGGGCGTGGTTGGCCTGATGATGGCGCAGATTATGGGCGTACGCGACAACGCCACGCTGGATCGCGCCTGCGATCTCGGTCTGGCGTTTCAGCTGACCAATATTGCGCGCGATATCGTTGAAGATGCTGAAGCGGGACGCTGCTATCTGCCCGCTGCGTGGCTGGCTGAAGAGGGGCTGACCCGAGAGAATCTCGCCGATCCGCAAAATCGCAAGGCATTAAGCCGCGTCGCCCGTCGGCTGGTGGAAACGGCGGAGCCCTATTATCGATCGGCGTCGGCTGGCCTGCCGGGTTTACCGCTGCGTTCAGCGTGGGCGATTGCTACCGCGCAGCAGGTCTATCGTAAAATCGGTATGAAGGTGGTTCAGGCGGGTTCACAGGCGTGGGAGCAACGCCAGTCCACCAGCACGCCAGAGAAACTGGCACTGCTGGTGGCGGCATCGGGTCAGGCGGTTACTTCCCGGGTGGCGCGTCACGCTCCACGCTCAGCTGATCTCTGGCAGCGCCCCGTTTAAGGATCC |
