# Supplementary material for: An electroporation-free method based on Red recombineering for markerless deletion and genomic replacement in the Escherichia coli DH1 genome
Source: PLoS One. 2017 Oct 24;12(10):e0186891. doi: 10.1371/journal.pone.0186891 (PMC5655456; doi:10.1371/journal.pone.0186891)
Supplement: S3 Table — (DOCX) [file pone.0186891.s009.docx]

**S3 Table. Primers used in this study.**

| Primer | Sequence |
| --- | --- |
| 1L-5 | GCCAAGCTTGGCTGGTGAAAGAGTCCC |
| 1L-3d | GCCGAATTCTTTCAAGCCTTATCAGCGGACTTTTTTTTGCAGTTTATGGTCTATTGCATGATTTGCGGGATGATAATTGC |
| 1R-5 | GCCGGATCCATGCAATAGACCATAAACTGCA |
| 1R-3 | GCCCTCGAGACCAGGTTAGCACCACGA |
| 2L-5 | GCCAAGCTTAATGACTATGCGTAACTTCG |
| 2L-3d | GCCGAATTCAGCGCACGTCACCGCAGCATCGTCATCAGCTCCATGGGAGAACGATGCTGCTAGCAGATTTGCCTTATAGCG |
| 2R-5 | GCCAGATCTAGCAGCATCGTTCTCCCAT |
| 2R-3 | GCCCTCGAGTTAGGGCTGAGCAGTATGA |
| 5L-5 | GCCAAGCTTAGGTGATATGCCTGATAGTAA |
| 5L-3d | GCCCAATTGGAGATTCCAATTATCGCGTCCAGCATGGTGTATCAGTGAGCTGCTTAGTTCATTAATGACAACCTTTTACGAG |
| 5R-5 | GCCGGATCCAACTAAGCAGCTCACTGATAC |
| 5R-3 | GCCCTCGAGCAGAACCGACTATTATCCATTA |
| 7L-5 | GCCAAGCTTCGCTGAAAGAGGCGATGG |
| 7L-3d | GCCGAATTCGCGACGAAAAATATGCGCGACATGCCAGCTATCCTTGCGGTTAAGTACATGATGAACGCGTCTTTGCTC |
| 7R-5 | GCCGGATCCATGTACTTAACCGCAAGGATA |
| 7R-3 | GCCCTCGAGCGCTTCAAAGGTGTCGTC |
| 8L-5 | GCCAAGCTTAGGCTACCGTCCGCAGTT |
| 8L-3d | GCCGAATTCTACTTAACCACCGAACGCAAGCGTATTGCTAGTAAAGTAAACAGCCTTCGTAATCCCTTGTGACGTAAAAAC |
| 8R-5 | GCCGGATCCCGAAGGCTGTTTACTTTACTA |
| 8R-3 | GCCCTCGAGGACGCATCAGAGCATCAAC |
| 8L-3i | GCCGAATTCTCTAAACGGGTCTTGAGGGGTTTTTTGCTGAAAGGAGGAACTATATCCGGTAATCCCTTGTGACGTAAAAAC |
| 8R-5i | GCCGGATCCGGTATGGTGGCAGGCCCCGTGGCCGGGGGACTGTTGGGCGCCATCTCCTTCGAAGGCTGTTTACTTTACTA |
| 8rL-3i | GCCGAATTCGGTATGGTGGCAGGCCCCGTGGCCGGGGGACTGTTGGGCGCCATCTCCTTTAATCCCTTGTGACGTAAAAAC |
| 8rR-5i | GCCGGATCCTCTAAACGGGTCTTGAGGGGTTTTTTGCTGAAAGGAGGAACTATATCCGGCGAAGGCTGTTTACTTTACTAG |
| 19L-5 | GCCAAGCTTACACCGCTTGGCGAGATT |
| 19L-3d | GCCGAATTCAGCATCCCGTGCTATGTTATTGACACACAAAAGCGTTGAGGAACAGTGAGCCATAAATTCAAGCGCAGTG |
| 19R-5 | GCCGGATCCCTCACTGTTCCTCAACGCTT |
| 19R-3 | GCCCTCGAGTGCCAGACAGCATCTACCC |
| 23L-5 | GCCAAGCTTGGATGCTGATTCCCTGTTCT |
| 23L-3i | GCCGAATTCTCTAAACGGGTCTTGAGGGGTTTTTTGCTGAAAGGAGGAACTATATCCGGGATAGTCGTACACAGTTCAC |
| 23R-5i | GCCGGATCCGGTATGGTGGCAGGCCCCGTGGCCGGGGGACTGTTGGGCGCCATCTCCTTCGCCGTTAAAACCTCCCTA |
| 23-R3 | GCCCTCGAGATCGCATCGTGCTGAAGG |
| 23rL-3i | GCCGAATTCGGTATGGTGGCAGGCCCCGTGGCCGGGGGACTGTTGGGCGCCATCTCCTTGATAGTCGTACACAGTTCAC |
| 23rR-5i | GCCGGATCCTCTAAACGGGTCTTGAGGGGTTTTTTGCTGAAAGGAGGAACTATATCCGGCGCCGTTAAAACCTCCCTA |
| 55L-5 | GCCAAGCTTCAACATCGCCAGCTTCATC |
| 55L-3d | GCCGAATTCAGAGACCTCGTGTGCTACCATCATCATTACTCAAGGTGGTCTCCCTATAGTGAGTCGT |
| 55R-5 | GCCGGATCCGATGTACGTTGATATCGGTT |
| 55R-3 | GCCCTCGAGGACAGATCGGAATGGCAGA |
| 57-L5 | GCCAAGCTTACCGCATCGTGAGCATCT |
| 57-L3i | GCCGAATTCTCTAAACGGGTCTTGAGGGGTTTTTTGCTGAAAGGAGGAACTATATCCGGTGCAAAAGCTAAAACAGCAG |
| 57-R5i | GCCGGATCCGGTATGGTGGCAGGCCCCGTGGCCGGGGGACTGTTGGGCGCCATCTCCTTCAATACGTAATTATCTTACCAGC |
| 57-R3 | GCCCTCGAGTCGACGGCGTAACCGATC |
| 63L-5 | GCCAAGCTTTGAATACGGCGAGCAACG |
| 63L-3d | GCCGAATTCATTGCTTTCTTTTTTGGCGTAAGCGTAAGATGCTTCATCTGGTTTAAACCAGCTTGAGGTGGTAACGC |
| 63R-5 | GCCGGATCCGGTTTAAACCAGATGAAGCA |
| 63R-3 | GCCCTCGAGTCTAACGGTCAGGGTAAAGC |
| 64-L5 | GCCAAGCTTCGCACGCCAAACCCATTA |
| 64-L3i | GCCGAATTCTCTAAACGGGTCTTGAGGGGTTTTTTGCTGAAAGGAGGAACTATATCCGGCCGCACTTACAGCAACGC |
| 64-R5i | GCCGGATCCGGTATGGTGGCAGGCCCCGTGGCCGGGGGACTGTTGGGCGCCATCTCCTTTTTATACTCCTGCGTCCTGT |
| 64-R3 | GCCCTCGAGGAACGGCACATCACTTTTAT |
| 1-0 | AACTGCGTGGCTTGCTGG |
| 1-1 | GCCTTAGGGAATTAGTTGAT |
| 2-0 | CTCGCCGTCAGGGAGCA |
| 2-1 | ATGCTGATTGGCGGTCTG |
| 5-0 | GAAAGGCTGAGGATGAGTG |
| 5-1 | CATTACCATTTATCACAACCC |
| 7-0 | CGCCGAAGCGACCAATGC |
| 7-1 | TCATCCGCTGCGAATCCC |
| 8-0 | GTGGTCAGGTACTGGCTAA |
| 8-1 | GAAACGCCGTCTTCTGTG |
| 19-0 | CGCATTGATGAACGACTGG |
| 19-1 | AACTGGCTGGTCAGGGTC |
| 23-0 | GTAGGGGTTTACCTGGCC |
| 23-1 | TTGCGTCCAGCCCGTTTT |
| 55-0 | AAACGGGTGAAGAACTACTG |
| 55-1 | TCCAGCCAATCCCGACAC |
| 57-0 | GCGTGTTCTACTTCTGCC |
| 57-1 | GACGAGAAAAGCGACGGT |
| 63-0 | TTGCCTCTGATTTCTCGTC |
| 63-1 | AAGCCGTGGTCTGATTGG |
| 64-0 | ACCGCACGACGCAGATTC |
| 64-1 | ATGTCTCGGTGGACTGAAT |
| Cm-1^#^ | ACCGTAACACGCCACATC |
| M13F | GTTGTAAAACGACGGCCAGT |
| M13R | CAGGAAACAGCTATGACC |
| T7 | TAATACGACTCACTATAGGG |
| T7t | TGCTAGTTATTGCTCAGCGG |
| KC5 | CGAATAAATACCTGTGACGGAAGATCACTTCGCAGAATAAATAAATCCTGGAGTTGGTAGCTCTTGATC |
| KC3 | CCAGCAATAGACATAAGCGGCTATTTAACGACCCTGCCCTGAACCGACGACAGGTGGCACTTTTCGG |
| CRT4 | TATCTCGAGTAAGGAGGATATTTAGATGAATAGAACTACAGTAATTGGCGC |
| CRT5 | GGAATTCGGATCCTTAAACGGGGCGCTGCCA |
| CRT6 | CATCTGGGAACCGGCATAG |
| CRT7 | CTATGCCGGTTCCCAGATG |
| CRT8 | CGCGGATCCTCAAACAGTTTCAC |
| CRT9 | TTTGGAAGATCTGGGCGGC |
| BRIS5 | GGAATTCCAGGGCCCTAGGGATAACAGGGTAATGTTTGACAGCTTATCATCG |
| BRIS3 | GGAATTCCGCGGCCGCATTACCCTGTTATCCCTAACGTGAGTTTTCGTTCCAC |
| E3A | GGTGCATGCGGGCCCAAGGAGATGGCGCCCAACA |
| E3N | GGGAATTCTCATATCATCGATGCGGCCGCCGGATATAGTTCCTCCTT |

^#^The sequence of primer Cm-1 is complementary to the *cat* gene sequence. In the first step of genomic modification, the colonies can be verified by PCR using primers X-0 (Y-0) and Cm-1. Positive colonies were approximately 1.3 kb (0.5 kb of the left homology region and part of the *cat* gene), and negative colonies did not have a DNA band.
