# Supplementary material for: An electroporation-free method based on Red recombineering for markerless deletion and genomic replacement in the Escherichia coli DH1 genome
Source: PLoS One. 2017 Oct 24;12(10):e0186891. doi: 10.1371/journal.pone.0186891 (PMC5655456; doi:10.1371/journal.pone.0186891)
Supplement: S4 Table — (DOCX) [file pone.0186891.s010.docx]

**S4 Table.** **Nonessential sequences in the *Escherichia coli* DH1 genome (Our laboratory).**

| Region | Left | Right | Length | Region | Left | Right | Length |  |  |
| --- | --- | --- | --- | --- | --- | --- | --- | --- | --- |
| **1** | **6254** | **16165** | **9912 bp** | 33 | 1967356 | 1968554 | 1199 bp |  |  |
| **2** | **17432** | **28605** | **11174 bp** | 34 | 2225717 | 2255844 | 30128 bp |  |  |
| 3 | 113902 | 122163 | 8262 bp | 35 | 2344852 | 2350586 | 5735 bp |  |  |
| 4 | 162082 | 163523 | 1442 bp | 36 | 2396224 | 2478151 | 81928 bp |  |  |
| **5** | **230444** | **232865** | **2422 bp** | 37 | 2478889 | 2480087 | 1199 bp |  |  |
| 6 | 246357 | 265152 | 18796 bp | 38 | 2481055 | 2490788 | 9734 bp |  |  |
| **7** | **299113** | **303017** | **3905 bp** | 39 | 2654571 | 2682872 | 28302 bp |  |  |
| **8** | **413909** | **430258** | **16350 bp** | 40 | 2738753 | 2750342 | 11590 bp |  |  |
| 9 | 512728 | 522538 | 9811 bp | 41 | 2782237 | 2839088 | 56852 bp |  |  |
| 10 | 692073 | 698988 | 6916 bp | 42 | 2971825 | 2990206 | 18382 bp |  |  |
| 11 | 710322 | 717649 | 7328 bp | 43 | 2997731 | 3013566 | 15836 bp |  |  |
| 12 | 747456 | 773166 | 25711 bp | **44** | **3033115** | **3048652** | **15538 bp** |  |  |
| 13 | 850854 | 897880 | 47027 bp | 45 | 3141612 | 3152384 | 10773 bp |  |  |
| 14 | 972413 | 972712 | 300 bp | 46 | 3191831 | 3206101 | 14271 bp |  |  |
| 15 | 1053021 | 1057967 | 4947 bp | 47 | 3271785 | 3305875 | 34091 bp |  |  |
| 16 | 1073133 | 1078972 | 5840 bp | 48 | 3319627 | 3334338 | 14712 bp |  |  |
| 17 | 1082390 | 1088106 | 5717 bp | 49 | 3337131 | 3351812 | 14682 bp |  |  |
| 18 | 1092514 | 1127604 | 35091 bp | 50 | 3466141 | 3481012 | 14872 bp |  |  |
| **19** | **1359035** | **1367343** | **8309 bp** | 51 | 3482313 | 3609066 | 126754 bp |  |  |
| 20 | 1368722 | 1378204 | 9483 bp | 52 | 3617632 | 3627077 | 9446 bp |  |  |
| 21 | 1400796 | 1410429 | 9634 bp | 53 | 3697930 | 3704311 | 6382 bp |  |  |
| 22 | 1449181 | 1459914 | 10734 bp | 54 | 3850815 | 3855989 | 5175 bp |  |  |
| **23** | **1487032** | **1496241** | **9210 bp** | **55** | **3912835** | **4017200** | **104366 bp** |  |  |
| 24 | 1586681 | 1590460 | 3780 bp | 56 | 4079174 | 4079952 | 779 bp |  |  |
| 25 | 1617562 | 1625158 | 7597 bp | 57 | 4090884 | 4104502 | 13619 bp |  |  |
| 26 | 1651204 | 1680385 | 29182 bp | **58** | **4184313** | **4200717** | **16405 bp** |  |  |
| 27 | 1693213 | 1734787 | 41575 bp | 59 | 4202525 | 4215059 | 12535 bp |  |  |
| 28 | 1740342 | 1776661 | 36320 bp | 60 | 4262189 | 4278565 | 16377 bp |  |  |
| 29 | 1797471 | 1811875 | 14405 bp | 61 | 4364214 | 4374696 | 10483 bp |  |  |
| 30 | 1836677 | 1847604 | 10928 bp | 62 | 4413943 | 4427477 | 13535 bp |  |  |
| 31 | 1852844 | 1884391 | 31548 bp | **63** | **4434265** | **4454029** | **19765 bp** |  |  |
| 32 | 1887977 | 1915329 | 27353 bp | 64 | 4575092 | 4587316 | 12225 bp |  |  |
