# Supplementary material for: Correction: An electroporation-free method based on Red recombineering for markerless deletion and genomic replacement in the Escherichia coli DH1 genome
Source: PLoS One. 2020 Feb 6;15(2):e0229072. doi: 10.1371/journal.pone.0229072 (PMC7004325; doi:10.1371/journal.pone.0229072)
Supplement: S2 Table — (DOCX) [file pone.0229072.s001.docx]

**S2 Table. Sequences of *I-SceI*, *I-CreI*, and *GC* cassette.**

|  | DNA sequence |
| --- | --- |
| *pSNA (I-SceI )* | GTTCCCAACGATCAAGGCGAGTTACATGATCCCCCATGTTGTGCAAAAAAGCGGTTAGCTCCTTCGGTCCTCCGATCGTTGTCAGAAGTAAGTTGGCCGCAGTGTTATCACTCATGGTTATGGCAGCACTGCATAATTCTCTTACTGTCATGCCATCCGTAAGATGCTTTTCTGTGACTGGTGAGTACTCAACCAAGTCATTCTGAGAATAGTGTATGCGGCGACCGAGTTGCTCTTGCCCGGCGTCAATACGGGATAATACCGCGCCACATAGCAGAACTTTAAAAGTGCTCATCATTGGAAAACGTTCTTCGGGGCGAAAACTCTCAAGGATCTTACCGCTGTTGAGATCCAGTTCGATGTAACCCACTCGTGCACCCAACTGATCTTCAGCATCTTTTACTTTCACCAGCGTTTCTGGGTGAGCAAAAACAGGAAGGCAAAATGCCGCAAAAAAGGGAATAAGGGCGACACGGAAATGTTGAATACTCATACTCTTCCTTTTTCAATATTATTGAAGCATTTATCAGGGTTATTGTCTCATGAGCGGATACATATTTGAATGTATTTAGAAAAATAAACAAATAGGGGTTCCGCGCACATTTCCCCGAAAAGTGTCGTCGGTTCAGGGCAGGGTCGTTAAATAGCCGCTTATGTCTATTGCTGGTCTCGGTACCCGGGGATCCTCTAGAGTCGACCTGCATAATGTGCCTGTCAAATGGACGAAGCAGGGATTCTGCAAACCCTATGCTACTCCGTCAAGCCGTCAATTGTCTGATTCGTTACCAATTATGACAACTTGACGGCTACATCATTCACTTTTTCTTCACAACCGGCACGGAACTCGCTCGGGCTGGCCCCGGTGCATTTTTTAAATACCCGCGAGAAATAGAGTTGATCGTCAAAACCAACATTGCGACCGACGGTGGCGATAGGCATCCGGGTGGTGCTCAAAAGCAGCTTCGCCTGGCTGATACGTTGGTCCTCGCGCCAGCTTAAGACGCTAATCCCTAACTGCTGGCGGAAAAGATGTGACAGACGCGACGGCGACAAGCAAACATGCTGTGCGACGCTGGCGATATCAAAATTGCTGTCTGCCAGGTGATCGCTGATGTACTGACAAGCCTCGCGTACCCGATTATCCATCGGTGGATGGAGCGACTCGTTAATCGCTTCCATGCGCCGCAGTAACAATTGCTCAAGCAGATTTATCGCCAGCAGCTCCGAATAGCGCCCTTCCCCTTGCCCGGCGTTAATGATTTGCCCAAACAGGTCGCTGAAATGCGGCTGGTGCGCTTCATCCGGGCGAAAGAACCCCGTATTGGCAAATATTGACGGCCAGTTAAGCCATTCATGCCAGTAGGCGCGCGGACGAAAGTAAACCCACTGGTGATACCATTCGCGAGCCTCCGGATGACGACCGTAGTGATGAATCTCTCCTGGCGGGAACAGCAAAATATCACCCGGTCGGCAAACAAATTCTCGTCCCTGATTTTTCACCACCCCCTGACCGCGAATGGTGAGATTGAGAATATAACCTTTCATTCCCAGCGGTCGGTCGATAAAAAAATCGAGATAACCGTTGGCCTCAATCGGCGTTAAACCCGCCACCAGATGGGCATTAAACGAGTATCCCGGCAGCAGGGGATCATTTTGCGCTTCAGCCATACTTTTCATACTCCCGCCATTCAGAGAAGAAACCAATTGTCCATATTGCATCAGACATTGCCGTCACTGCGTCTTTTACTGGCTCTTCTCGCTAACCAAACCGGTAACCCCGCTTATTAAAAGCATTCTGTAACAAAGCGGGACCAAAGCCATGACAAAAACGCGTAACAAAAGTGTCTATAATCACGGCAGAAAAGTCCACATTGATTATTTGCACGGCGTCACACTTTGCTATGCCATAGCATTTTTATCCATAAGATTAGCGGATCCTACCTGACGCTTTTTATCGCAACTCTCTACTGTTTCTCCATACCCGTTTTTTTGGGCTAGCAGGAGGGTACCTATATGCATATGAAAATCATCAAAAAAAACCAGGTAATGAACCTCGGTCCGAACTCTAAACTGCTGAAAGAATACAAATCCCAGCTGATCGAACTGAACATCGAACAGTTCGAAGCAGGTATCGGTCTGATCCTGGGTGATGCTTACATCCGTTCTCGTGATGAAGGTAAAACCTACTGTATGCAGTTCGAGTGGAAAAACAAAGCATACATGGACCACGTATGTCTGCTGTACGATCAGTGGGTACTGTCCCCGCCGCACAAAAAAGAACGTGTTAACCACCTGGGTAACCTGGTAATCACCTGGGGCGCCCAGACTTTCAAACACCAAGCATTCAACAAACTGGCTAACCTGTTCATCGTTAACAACAAAAAAACCATCCCGAACAACCTGGTTGAAAACTACCTGACCCCGATGTCTCTGGCATACTGGTTCATGGATGATGGTGGTAAATGGGATTACAACAAAAACTCTACCAACAAATCGATCGTACTGAACACCCAGTCTTTCACTTTCGAAGAAGTAGAATACCTGGTTAAGGGTCTGCGTAACAAATTCCAACTGAACTGTTACGTAAAAATCAACAAAAACAAACCGATCATCTACATCGATTCTATGTCTTACCTGATCTTCTACAACCTGATCAAACCGTACCTGATCCCGCAGATGATGTACAAACTGCCGAACACTATCTCCTCCGAAACTTTCCTGAAATAAGCTAGAAGATGTTTCGTGAAGCCGTCGACGCTTATAAAAAATGGATATTAATACTGAAACTGAGATCAAGCAAAAGCATTCACTAACCCCCTTTCCTGTTTTCCTAATCAGCCCGGCATTTCGCGGGCGATATTTTCACAGCTATTTCAGGAGTTCAGCCATGAACGCTTATTACATTCAGGATCGTCTTGAGGCTCAGAGCTGGGCGCGTCACTACCAGCAGCTCGCCCGTGAAGAGAAAGAGGCAGAACTGGCAGACGACATGGAAAAAGGCCTGCCCCAGCACCTGTTTGAATCGCTATGCATCGATCATTTGCAACGCCACGGGGCCAGCAAAAAATCCATTACCCGTGCGTTTGATGACGATGTTGAGTTTCAGGAGCGCATGGCAGAACACATCCGGTACATGGTTGAAACCATTGCTCACCACCAGGTTGATATTGATTCAGAGGTATAAAACGAATGAGTACTGC  ACTCGCAACGCTGGCTGGGAAGCTGGCTGAACGTGTCGGCATGGATTCTGTCGACCCACAGGAACTGATCACCACTCTTCGCCAGACGGCATTTAAAGGTGATGCCAGCGATGCGCAGTTCATCGCATTACTGATCGTTGCCAACCAGTACGGCCTTAATCCGTGGACGAAAGAAATTTACGCCTTTCCTGATAAGCAGAATGGCATCGTTCCGGTGGTGGGCGTTGATGGCTGGTCCCGCATCATCAATGAAAACCAGCAGTTTGATGGCATGGACTTTGAGCAGGACAATGAATCCTGTACATGCCGGATTTACCGCAAGGACCGTAATCATCCGATCTGCGTTACCGAATGGATGGATGAATGCCGCCGCGAACCATTCAAAACTCGCGAAGGCAGAGAAATCACGGGGCCGTGGCAGTCGCATCCCAAACGGATGTTACGTCATAAAGCCATGATTCAGTGTGCCCGTCTGGCCTTCGGATTTGCTGGTATCTATGACAAGGATGAAGCCGAGCGCATTGTCGAAAATACTGCATACACTGCAGAACGTCAGCCGGAACGCGACATCACTCCGGTTAACGATGAAACCATGCAGGAGATTAACACTCTGCTGATCGCCCTGGATAAAACATGGGATGACGACTTATTGCCGCTCTGTTCCCAGATATTTCGCCGCGACATTCGTGCATCGTCAGAACTGACACAGGCCGAAGCAGTAAAAGCTCTTGGATTCCTGAAACAGAAAGCCGCAGAGCAGAAGGTGGCAGCATGACACCGGACATTATCCTGCAGCGTACCGGGATCGATGTGAGAGCTGTCGAACAGGGGGAAGATGCGTGGCACAAATTACGGCTCGGCGTCATCACCGCTTCAGAAGTTCACAACGTGATAGCAAAACCCCGCTCCGGAAAGAAGTGGCCTGACATGAAAATGTCCTACTTCCACACCCTGCTTGCTGAGGTTTGCACCGGTGTGGCTCCGGAAGTTAACGCTAAAGCACTGGCCTGGGGAAAACAGTACGAGAACGACGCCAGAACCCTGTTTGAATTCACTTCCGGCGTGAATGTTACTGAATCCCCGATCATCTATCGCGACGAAAGTATGCGTACCGCCTGCTCTCCCGATGGTTTATGCAGTGACGGCAACGGCCTTGAACTGAAATGCCCGTTTACCTCCCGGGATTTCATGAAGTTCCGGCTCGGTGGTTTCGAGGCCATAAAGTCAGCTTACATGGCCCAGGTGCAGTACAGCATGTGGGTGACGCGAAAAAATGCCTGGTACTTTGCCAACTATGACCCGCGTATGAAGCGTGAAGGCCTGCATTATGTCGTGATTGAGCGGGATGAAAAGTACATGGCGAGTTTTGACGAGATCGTGCCGGAGTTCATCGAAAAAATGGACGAGGCACTGGCTGAAATTGGTTTTGTATTTGGGGAGCAATGGCGATGACGCATCCTCACGATAATAAGCTTCCTGCTGAACATCAAAGGCAAGAAAACATCTGTTGTCAAAGACAGCATCCTTGAACAAGGACAATTAACAGTTAACAAATAAAAACGCAAAAGAAAATGCCGATATTGACTACCGGAAGCAGTGTGACCGTGTGCTTCTCAAATGCCTGATTCAGGCTGTCTATGTGTGACTGTTGAGCTGTAACAAGTTGTCTCAGGTGTTCAATTTCATGTTCTAGTTGCTTTGTTTTACTGGTTTCACCTGTTCTATTAGGTGTTACATGCTGTTCATCTGTTACATTGTCGATCTGTTCATGGTGAACAGCTTTAAATGCACCAAAAACTCGTAAAAGCTCTGATGTATCTATCTTTTTTACACCGTTTTCATCTGTGCATATGGACAGTTTTCCCTTTGATATGTAACGGTGAACAGTTGTTCTACTTTTGTTTGTTAGTCTTGATGCTTCACTGATAGATACAAGAGCCATAAGAACCTCAGATCCTTCCGTATTTAGCCAGTATGTTCTCTAGTGTGGTTCGTTGTTTTTGCGTGAGCCATGAGAACGAACCATTGAGATCATACTTACTTTGCATGTCACTCAAAAATTTTGCCTCAAAACTGGTGAGCTGAATTTTTGCAGTTAAAGCATCGTGTAGTGTTTTTCTTAGTCCGTTATGTAGGTAGGAATCTGATGTAATGGTTGTTGGTATTTTGTCACCATTCATTTTTATCTGGTTGTTCTCAAGTTCGGTTACGAGATCCATTTGTCTATCTAGTTCAACTTGGAAAATCAACGTATCAGTCGGGCGGCCTCGCTTATCAACCACCAATTTCATATTGCTGTAAGTGTTTAAATCTTTACTTATTGGTTTCAAAACCCATTGGTTAAGCCTTTTAAACTCATGGTAGTTATTTTCAAGCATTAACATGAACTTAAATTCATCAAGGCTAATCTCTATATTTGCCTTGTGAGTTTTCTTTTGTGTTAGTTCTTTTAATAACCACTCATAAATCCTCATAGAGTATTTGTTTTCAAAAGACTTAACATGTTCCAGATTATATTTTATGAATTTTTTTAACTGGAAAAGATAAGGCAATATCTCTTCACTAAAAACTAATTCTAATTTTTCGCTTGAGAACTTGGCATAGTTTGTCCACTGGAAAATCTCAAAGCCTTTAACCAAAGGATTCCTGATTTCCACAGTTCTCGTCATCAGCTCTCTGGTTGCTTTAGCTAATACACCATAAGCATTTTCCCTACTGATGTTCATCATCTGAACGTATTGGTTATAAGTGAACGATACCGTCCGTTCTTTCCTTGTAGGGTTTTCAATCGTGGGGTTGAGTAGTGCCACACAGCATAAAATTAGCTTGGTTTCATGCTCCGTTAAGTCATAGCGACTAATCGCTAGTTCATTTGCTTTGAAAACAACTAATTCAGACATACATCTCAATTGGTCTAGGTGATTTTAATCACTATACCAATTGAGATGGGCTAGTCAATGATAATTACTAGTCCTTTTCCTTTGAGTTGTGGGTATCTGTAAATTCTGCTAGACCTTTGCTGGAAAACTTGTAAATTCTGCTAGACCCTCTGTAAATTCCGCTAGACCTTTGTGTGTTTTTTTTGTTTATATTCAAGTGGTTATAATTTATAGAATAAAGAAAGAATAAAAAAAGATAAAAAGAATAGATCCCAGCCCTGTGTATAACTCACTACTTTAGTCAGTTCCGCAGTATTACAAAAGGATGTCGCAAACGCTGTTTGCTCCTCTACAAAACAGACCTTAAAACCCTAAAGGCTTAAGTAGCACCCTCGCAAGCTCGGGCAAATCGCTGAATATTCCTTTTGTCTCCGACCATCAGGCACCTGAGTCGCTGTCTTTTTCGTGACATTCAGTTCGCTGCGCTCACGGCTCTGGCAGTGAATGGGGGTAAATGGCACTACAGGCGCCTTTTATGGATTCATGCAAGGAAACTACCCATAATACAAGAAAAGCCCGTCACGGGCTTCTCAGGGCGTTTTATGGCGGGTCTGCTATGTGGTGCTATCTGACTTTTTGCTGTTCAGCAGTTCCTGCCCTCTGATTTTCCAGTCTGACCACTTCGGATTATCCCGTGACAGGTCATTCAGACTGGCTAATGCACCCAGTAAGGCAGCGGTATCATCAACAGGCTTACCCGTCTTACTGTCGGGGATCGACGCTCTCCCTTATGCGACTCCTGCACCTTTCGTCTTCGAATAAATACCTGTGACGGAAGATCACTTCGCAGAATAAATAAATCCTGGTCTGACGCTCAGTGGAACGAAAACTCACGTTAAGGGATTTTGGTCATGAGATTATCAAAAAGGATCTTCACCTAGATCCTTTTAAATTAAAAATGAAGTTTTAAATCAATCTAAAGTATATATGAGTAAACTTGGTCTGACAGTTACCAATGCTTAATCAGTGAGGCACCTATCTCAGCGATCTGTCTATTTCGTTCATCCATAGTTGCCTGACTCCCCGTCGTGTAGATAACTACGATACGGGAGGGCTTACCATCTGGCCCCAGTGCTGCAATGATACCGCGAGACCCACGCTCACCGGCTCCAGATTTATCAGCAATAAACCAGCCAGCCGGAAGGGCCGAGCGCAGAAGTGGTCCTGCAACTTTATCCGCCTCCATCCAGTCTATTAATTGTTGCCGGGAAGCTAGAGTAAGTAGTTCGCCAGTTAATAGTTTGCGCAACGTTGTTGCCATTGCTGCAGGCATCGTGGTGTCACGCTCGTCGTTTGGTATGGCTTCATTCAGCTCCG |
| *pCNA (I-CreI)* | TCTAGCGAAAAGATGTTTCGTGAAGCCGTCGACGCTTATAAAAAATGGATATTAATACTGAAACTGAGATCAAGCAAAAGCATTCACTAACCCCCTTTCCTGTTTTCCTAATCAGCCCGGCATTTCGCGGGCGATATTTTCACAGCTATTTCAGGAGTTCAGCCATGAACGCTTATTACATTCAGGATCGTCTTGAGGCTCAGAGCTGGGCGCGTCACTACCAGCAGCTCGCCCGTGAAGAGAAAGAGGCAGAACTGGCAGACGACATGGAAAAAGGCCTGCCCCAGCACCTGTTTGAATCGCTATGCATCGATCATTTGCAACGCCACGGGGCCAGCAAAAAATCCATTACCCGTGCGTTTGATGACGATGTTGAGTTTCAGGAGCGCATGGCAGAACACATCCGGTACATGGTTGAAACCATTGCTCACCACCAGGTTGATATTGATTCAGAGGTATAAAACGAATGAGTACTGCACTCGCAACGCTGGCTGGGAAGCTGGCTGAACGTGTCGGCATGGATTCTGTCGACCCACAGGAACTGATCACCACTCTTCGCCAGACGGCATTTAAAGGTGATGCCAGCGATGCGCAGTTCATCGCATTACTGATCGTTGCCAACCAGTACGGCCTTAATCCGTGGACGAAAGAAATTTACGCCTTTCCTGATAAGCAGAATGGCATCGTTCCGGTGGTGGGCGTTGATGGCTGGTCCCGCATCATCAATGAAAACCAGCAGTTTGATGGCATGGACTTTGAGCAGGACAATGAATCCTGTACATGCCGGATTTACCGCAAGGACCGTAATCATCCGATCTGCGTTACCGAATGGATGGATGAATGCCGCCGCGAACCATTCAAAACTCGCGAAGGCAGAGAAATCACGGGGCCGTGGCAGTCGCATCCCAAACGGATGTTACGTCATAAAGCCATGATTCAGTGTGCCCGTCTGGCCTTCGGATTTGCTGGTATCTATGACAAGGATGAAGCCGAGCGCATTGTCGAAAATACTGCATACACTGCAGAACGTCAGCCGGAACGCGACATCACTCCGGTTAACGATGAAACCATGCAGGAGATTAACACTCTGCTGATCGCCCTGGATAAAACATGGGATGACGACTTATTGCCGCTCTGTTCCCAGATATTTCGCCGCGACATTCGTGCATCGTCAGAACTGACACAGGCCGAAGCAGTAAAAGCTCTTGGATTCCTGAAACAGAAAGCCGCAGAGCAGAAGGTGGCAGCATGACACCGGACATTATCCTGCAGCGTACCGGGATCGATGTGAGAGCTGTCGAACAGGGGGAAGATGCGTGGCACAAATTACGGCTCGGCGTCATCACCGCTTCAGAAGTTCACAACGTGATAGCAAAACCCCGCTCCGGAAAGAAGTGGCCTGACATGAAAATGTCCTACTTCCACACCCTGCTTGCTGAGGTTTGCACCGGTGTGGCTCCGGAAGTTAACGCTAAAGCACTGGCCTGGGGAAAACAGTACGAGAACGACGCCAGAACCCTGTTTGAATTCACTTCCGGCGTGAATGTTACTGAATCCCCGATCATCTATCGCGACGAAAGTATGCGTACCGCCTGCTCTCCCGATGGTTTATGCAGTGACGGCAACGGCCTTGAACTGAAATGCCCGTTTACCTCCCGGGATTTCATGAAGTTCCGGCTCGGTGGTTTCGAGGCCATAAAGTCAGCTTACATGGCCCAGGTGCAGTACAGCATGTGGGTGACGCGAAAAAATGCCTGGTACTTTGCCAACTATGACCCGCGTATGAAGCGTGAAGGCCTGCATTATGTCGTGATTGAGCGGGATGAAAAGTACATGGCGAGTTTTGACGAGATCGTGCCGGAGTTCATCGAAAAAATGGACGAGGCACTGGCTGAAATTGGTTTTGTATTTGGGGAGCAATGGCGATGACGCATCCTCACGATAATAAGCTTCCTGCTGAACATCAAAGGCAAGAAAACATCTGTTGTCAAAGACAGCATCCTTGAACAAGGACAATTAACAGTTAACAAATAAAAACGCAAAAGAAAATGCCGATATTGACTACCGGAAGCAGTGTGACCGTGTGCTTCTCAAATGCCTGATTCAGGCTGTCTATGTGTGACTGTTGAGCTGTAACAAGTTGTCTCAGGTGTTCAATTTCATGTTCTAGTTGCTTTGTTTTACTGGTTTCACCTGTTCTATTAGGTGTTACATGCTGTTCATCTGTTACATTGTCGATCTGTTCATGGTGAACAGCTTTAAATGCACCAAAAACTCGTAAAAGCTCTGATGTATCTATCTTTTTTACACCGTTTTCATCTGTGCATATGGACAGTTTTCCCTTTGATATGTAACGGTGAACAGTTGTTCTACTTTTGTTTGTTAGTCTTGATGCTTCACTGATAGATACAAGAGCCATAAGAACCTCAGATCCTTCCGTATTTAGCCAGTATGTTCTCTAGTGTGGTTCGTTGTTTTTGCGTGAGCCATGAGAACGAACCATTGAGATCATACTTACTTTGCATGTCACTCAAAAATTTTGCCTCAAAACTGGTGAGCTGAATTTTTGCAGTTAAAGCATCGTGTAGTGTTTTTCTTAGTCCGTTATGTAGGTAGGAATCTGATGTAATGGTTGTTGGTATTTTGTCACCATTCATTTTTATCTGGTTGTTCTCAAGTTCGGTTACGAGATCCATTTGTCTATCTAGTTCAACTTGGAAAATCAACGTATCAGTCGGGCGGCCTCGCTTATCAACCACCAATTTCATATTGCTGTAAGTGTTTAAATCTTTACTTATTGGTTTCAAAACCCATTGGTTAAGCCTTTTAAACTCATGGTAGTTATTTTCAAGCATTAACATGAACTTAAATTCATCAAGGCTAATCTCTATATTTGCCTTGTGAGTTTTCTTTTGTGTTAGTTCTTTTAATAACCACTCATAAATCCTCATAGAGTATTTGTTTTCAAAAGACTTAACATGTTCCAGATTATATTTTATGAATTTTTTTAACTGGAAAAGATAAGGCAATATCTCTTCACTAAAAACTAATTCTAATTTTTCGCTTGAGAACTTGGCATAGTTTGTCCACTGGAAAATCTCAAAGCCTTTAACCAAAGGATTCCTGATTTCCACAGTTCTCGTCATCAGCTCTCTGGTTGCTTTAGCTAATACACCATAAGCATTTTCCCTACTGATGTTCATCATCTGAACGTATTGGTTATAAGTGAACGATACCGTCCGTTCTTTCCTTGTAGGGTTTTCAATCGTGGGGTTGAGTAGTGCCACACAGCATAAAATTAGCTTGGTTTCATGCTCCGTTAAGTCATAGCGACTAATCGCTAGTTCATTTGCTTTGAAAACAACTAATTCAGACATACATCTCAATTGGTCTAGGTGATTTTAATCACTATACCAATTGAGATGGGCTAGTCAATGATAATTACTAGTCCTTTTCCTTTGAGTTGTGGGTATCTGTAAATTCTGCTAGACCTTTGCTGGAAAACTTGTAAATTCTGCTAGACCCTCTGTAAATTCCGCTAGACCTTTGTGTGTTTTTTTTGTTTATATTCAAGTGGTTATAATTTATAGAATAAAGAAAGAATAAAAAAAGATAAAAAGAATAGATCCCAGCCCTGTGTATAACTCACTACTTTAGTCAGTTCCGCAGTATTACAAAAGGATGTCGCAAACGCTGTTTGCTCCTCTACAAAACAGACCTTAAAACCCTAAAGGCTTAAGTAGCACCCTCGCAAGCTCGGGCAAATCGCTGAATATTCCTTTTGTCTCCGACCATCAGGCACCTGAGTCGCTGTCTTTTTCGTGACATTCAGTTCGCTGCGCTCACGGCTCTGGCAGTGAATGGGGGTAAATGGCACTACAGGCGCCTTTTATGGATTCATGCAAGGAAACTACCCATAATACAAGAAAAGCCCGTCACGGGCTTCTCAGGGCGTTTTATGGCGGGTCTGCTATGTGGTGCTATCTGACTTTTTGCTGTTCAGCAGTTCCTGCCCTCTGATTTTCCAGTCTGACCACTTCGGATTATCCCGTGACAGGTCATTCAGACTGGCTAATGCACCCAGTAAGGCAGCGGTATCATCAACAGGCTTACCCGTCTTACTGTCGGGGATCGACGCTCTCCCTTATGCGACTCCTGCACCTTTCGTCTTCGAATAAATACCTGTGACGGAAGATCACTTCGCAGAATAAATAAATCCTGGTGTCCCTGGACGAAAGGGCCTCGTGATACGCCTATTTTTATAGGTTAATGTCATGATAATAATGGTTTCTTAGACGTCAGGTGGCACTTTTCGGGGAAATGTGCGCGGAACCCCTATTTGTTTATTTTTCTAAATACATTCAAATATGTATCCGCTCATGAGACAATAACCCTGATAAATGCTTCAATAATATTGAAAAAGGAAGAGTATGAGTATTCAACATTTCCGTGTCGCCCTTATTCCCTTTTTTGCGGCATTTTGCCTTCCTGTTTTTGCTCACCCAGAAACGCTGGTGAAAGTAAAAGATGCTGAAGATCAGTTGGGTGCACGAGTGGGTTACATCGAACTGGATCTCAACAGCGGTAAGATCCTTGAGAGTTTTCGCCCCGAAGAACGTTTTCCAATGATGAGCACTTTTAAAGTTCTGCTATGTGGCGCGGTATTATCCCGTATTGACGCCGGGCAAGAGCAACTCGGTCGCCGCATACACTATTCTCAGAATGACTTGGTTGAGTACTCACCAGTCACAGAAAAGCATCTTACGGATGGCATGACAGTAAGAGAATTATGCAGTGCTGCCATAACCATGAGTGATAACACTGCGGCCAACTTACTTCTGACAACGATCGGAGGACCGAAGGAGCTAACCGCTTTTTTGCACAACATGGGGGATCATGTAACTCGCCTTGATCGTTGGGAACCGGAGCTGAATGAAGCCATACCAAACGACGAGCGTGACACCACGATGCCTGTAGCAATGGCAACAACGTTGCGCAAACTATTAACTGGCGAACTACTTACTCTAGCTTCCCGGCAACAATTAATAGACTGGATGGAGGCGGATAAAGTTGCAGGACCACTTCTGCGCTCGGCCCTTCCGGCTGGCTGGTTTATTGCTGATAAATCTGGAGCCGGTGAGCGTGGGTCTCGCGGTATCATTGCAGCACTGGGGCCAGATGGTAAGCCCTCCCGTATCGTAGTTATCTACACGACGGGGAGTCAGGCAACTATGGATGAACGAAATAGACAGATCGCTGAGATAGGTGCCTCACTGATTAAGCATTGGTAACTGTCAGACCAAGTTTACTCATATATACTTTAGATTGCGTAATTTTTTTAAGGCAGTTATTGGTGCCCTTAAACGCCTGGTTGCTACGCCTGAATAAGTGATAATAAGCGGATGAATGGCAGAAATTCGAAAGCAAATTCGACCCGGTCGTCGGTTCAGGGCAGGGTCGTTAAATAGCCGCTTATGTCTATTGCTGGTCTCGGTACCCGGGGATCCTCTAGAGTCGACCTGCATAATGTGCCTGTCAAATGGACGAAGCAGGGATTCTGCAAACCCTATGCTACTCCGTCAAGCCGTCAATTGTCTGATTCGTTACCAATTATGACAACTTGACGGCTACATCATTCACTTTTTCTTCACAACCGGCACGGAACTCGCTCGGGCTGGCCCCGGTGCATTTTTTAAATACCCGCGAGAAATAGAGTTGATCGTCAAAACCAACATTGCGACCGACGGTGGCGATAGGCATCCGGGTGGTGCTCAAAAGCAGCTTCGCCTGGCTGATACGTTGGTCCTCGCGCCAGCTTAAGACGCTAATCCCTAACTGCTGGCGGAAAAGATGTGACAGACGCGACGGCGACAAGCAAACATGCTGTGCGACGCTGGCGATATCAAAATTGCTGTCTGCCAGGTGATCGCTGATGTACTGACAAGCCTCGCGTACCCGATTATCCATCGGTGGATGGAGCGACTCGTTAATCGCTTCCATGCGCCGCAGTAACAATTGCTCAAGCAGATTTATCGCCAGCAGCTCCGAATAGCGCCCTTCCCCTTGCCCGGCGTTAATGATTTGCCCAAACAGGTCGCTGAAATGCGGCTGGTGCGCTTCATCCGGGCGAAAGAACCCCGTATTGGCAAATATTGACGGCCAGTTAAGCCATTCATGCCAGTAGGCGCGCGGACGAAAGTAAACCCACTGGTGATACCATTCGCGAGCCTCCGGATGACGACCGTAGTGATGAATCTCTCCTGGCGGGAACAGCAAAATATCACCCGGTCGGCAAACAAATTCTCGTCCCTGATTTTTCACCACCCCCTGACCGCGAATGGTGAGATTGAGAATATAACCTTTCATTCCCAGCGGTCGGTCGATAAAAAAATCGAGATAACCGTTGGCCTCAATCGGCGTTAAACCCGCCACCAGATGGGCATTAAACGAGTATCCCGGCAGCAGGGGATCATTTTGCGCTTCAGCCATACTTTTCATACTCCCGCCATTCAGAGAAGAAACCAATTGTCCATATTGCATCAGACATTGCCGTCACTGCGTCTTTTACTGGCTCTTCTCGCTAACCAAACCGGTAACCCCGCTTATTAAAAGCATTCTGTAACAAAGCGGGACCAAAGCCATGACAAAAACGCGTAACAAAAGTGTCTATAATCACGGCAGAAAAGTCCACATTGATTATTTGCACGGCGTCACACTTTGCTATGCCATAGCATTTTTATCCATAAGATTAGCGGATCCTACCTGACGCTTTTTATCGCAACTCTCTACTGTTTCTCCATACCCGTTTTTTTGGGCTAGCTAAGAAGGAGATATACATATGTTATACCTGGCAGGTTTCGTGGACGGTGACGGTAGCATCATCGCTCAGATTAAACCAAACCAGTCTTATAAGTTTAAACATCAGCTGAGCTTGACCTTTCAGGTGACTCAAAAGACCCAGCGCCGTTGGTTTCTGGACAAACTAGTGGATGAAATTGGCGTTGGTTACGTACGTGATCGCGGTTCCGTTTCCGATTACATCTTAAGCGAAATCAAGCCGCTGCACAACTTCCTGACTCAACTGCAGCCGTTTCTGAAACTGAAACAGAAACAGGCAAACCTGGTTCTGAAAATTATCGAACAGCTGCCGTCTGCAAAAGAATCCCCGGACAAATTCCTGGAAGTTTGTACCTGGGTGGATCAGATTGCAGCTCTGAACGATTCTAAGACTCGTAAAACCACTTCTGAAACCGTTCGTGCTGTGCTGGACAGCCTGAGCGAGAAGAAGAAATCCTCCCCGGCGGCCGACTAA |
| *GC* cassette | CATATGGATAGATTTCAGCGTTTGATTGCCATGCTGAAGGAGGAAATTGCGAAACGTGCCGAAATTATCAACAAAGCCATTGAAGAGCTTCTGCCGGAACGTGAGCCGATTGGTCTCTACAAAGCCGCACGTCATCTGATCAAAGCAGGTGGCAAGCGTCTGCGTCCTGTAATCAGCCTCTTAGCAGTCGAAGCCCTTGGTAAAGACTACAGAAAGATTATCCCGGCTGCTGTCAGCATTGAAACAATCCACAACTTCACCCTCGTGCATGACGACATCATGGACCGTGACGAGATGCGTCGTGGTGTTCCGACTGTACACAGAGTTTATGGTGAAGCGACTGCCATTTTAGCAGGCGACACACTCTTTGCTGAAGCCTTCAAGCTGCTGACAAAGTGCGATGTTGAGAGCGAGGGTATCAGAAAAGCTACAGAAATGCTTTCGGACGTTTGCATTAAAATTTGCGAGGGTCAGTACTACGACATGAGCTTTGAGAAAAAGGAGAGCGTTTCCGAGGAGGAGTATCTCAGAATGGTCGAGCTGAAGACCGGTGTGCTGATTGCAGCTTCTGCAGCATTACCTGCGGTGCTTTTTGGTGAGAGCGAGGAAATTGTAAAGGCGCTGTGGGACTACGGTGTTCTTAGCGGTATTGGCTTCCAGATCCAGGACGACCTGCTTGACCTGACTGAGGAGACCGGTAAGGACTGGGGTAGCGACCTGCTTAAAGGTAAGAAAACCCTGATTGTCATTAAGGCGTTCGAAAAGGGTGTGAAGCTGAAGACATTTGGTAAGGAAAAGGCGGACGTCTCTGAGATTAGAGATGATATCGAAAAGTTAAGAGAGTGTGGTGCGATTGATTACGCTGCCAGCATGGCAAGAAAGATGGCTGAAGAGGCGAAAAGAAAGCTCGAAGTTCTGCCTGAAAGCAAAGCCAAGGAAACACTGCTGGAACTTACCGACTTCTTGGTTACAAGAAAAAAGTAACTCGAGTAAGGAGGATATTTAGATGAATAGAACTACAGTAATTGGCGCAGGCTTTGGTGGTCTGGCTCTGGCCATTCGCCTTCAGGCGTCAGGCGTTCCCACCCGACTGCTGGAGCAGCGTGACAAGCCGGGCGGCCGGGCTTATGTCTATCAGGATCAGGGCTTCACGTTTGATGCCGGCCCCACGGTAATCACCGATCCCAGCGCCATTGAAGAGCTGTTCACTCTGGCGGGTAAAAAGCTCTCTGACTATGTCGAGCTGATGCCGGTGAAGCCGTTTTATCGCCTCTGCTGGGAGTCCGGCAAGGTGTTCAGTTATGACAACGATCAGCCCGCGCTGGAAGCGCAGATTGCCGCATTTAATCCGCGTGACGTTGAAGGATATCGGCGCTTTCTGGCCTATTCCCGAGCGGTGTTTGCTGAAGGCTATCTGAAGCTTGGCACCGTGCCGTTTCTGTCATTCCGCGACATGCTGCGGGCCGCGCCTCAGCTGGCAAAACTTCAGGCATGGCGCAGCGTTTACAGCAAAGTGGCGAGCTACATTGAAGATGAGCATCTGCGTCAGGCCTTCTCTTTCCACTCACTGCTGGTGGGCGGAAATCCGTTTGCCACTTCCTCAATCTATACCCTGATTCATGCGCTGGAACGTGAATGGGGCGTCTGGTTCCCGCGCGGTGGCACGGGCGCGCTGGTGCAGGGCATGGTGAAACTGTTTGAGGATCTGGGCGGCGAAGTGGAGCTCAATGCCAGCGTTGCCCGGCTGGAGACCCAGGAAAACAGGATTACCGCGGTGCACCTGAAAGATGGCCGGGTCTTCCCGACCCGCGCGGTTGCCTCCAACGCAGATGTGGTTCACACCTACCGCGAACTGCTGAGCCAGCACCCCGCTTCGCAGGCGCAGGGACGGTCACTGCAGAACAAACGCATGAGTAACTCGCTGTTTGTGATCTATTTTGGCCTGAATCATCATCACGATCAGCTGGCGCACCACACGGTCTGCTTTGGTCCGCGCTATCGTGAGTTGATTGATGAAATCTTTAACAAAGATGGCCTGGCAGAGGACTTCTCGCTCTATCTGCACGCGCCCTGCGTGACCGATCCCTCACTGGCACCGGAAGGCTGCGGCAGCTACTACGTGCTGGCGCCGGTACCGCACCTCGGCACCGCTGATATCGACTGGGCCGTTGAAGGTCCGCGCCTGCGCGATCGCATTTTCGACTATCTGGAACAGCATTACATGCCGGGCCTGCGTAGCCAGTTGGTCACGCATCGCATCTTCACGCCGTTTGATTTCCGCGATGAGCTGAATGCGTATCAGGGCTCGGCCTTCTCAGTGGAGCCGATCCTGACGCAAAGCGCCTGGTTCCGGCCTCACAACCGCGATAAAAATATTAATAATCTCTATCTGGTCGGTGCTGGTACCCATCCTGGCGCGGGTATTCCAGGGGTGATTGGCTCGGCCAAGGCTACCGCAGGATTGATGCTGGAGGATCTGGCTTGAATAGTCCGTCACTGCTTGATCATGCCGTAGACACCATGGAGGTGGGATCGAAAAGCTTTGCCACCGCGTCAAAACTGTTTGATGCCAAAACCCGACGCAGCGTGCTGATGCTCTACGCCTGGTGCCGTCACTGTGATGATGTGATTGACGATCAGGTCCTGGGATTCAGCAACGATACGCCATCGCTGCAATCTGCCGAACAGCGCCTGGCGCAGCTGGAGATGAAAACGCGTCAGGCCTATGCCGGTTCCCAGATGCATGAGCCCGCCTTTGCGGCCTTTCAGGAGGTGGCAATGGCGCACGATATTCTGCCTGCTTACGCTTTTGATCATCTGGCGGGCTTTGCGATGGACGTGCATGAGACACGCTATCAGACGCTGGATGATACGCTGCGTTACTGTTACCACGTCGCGGGCGTGGTTGGCCTGATGATGGCGCAGATTATGGGCGTACGCGACAACGCCACGCTGGATCGCGCCTGCGATCTCGGTCTGGCGTTTCAGCTGACCAATATTGCGCGCGATATCGTTGAAGATGCTGAAGCGGGACGCTGCTATCTGCCCGCTGCGTGGCTGGCTGAAGAGGGGCTGACCCGAGAGAATCTCGCCGATCCGCAAAATCGCAAGGCATTAAGCCGCGTCGCCCGTCGGCTGGTGGAAACGGCGGAGCCCTATTATCGATCGGCGTCGGCTGGCCTGCCGGGTTTACCGCTGCGTTCAGCGTGGGCGATTGCTACCGCGCAGCAGGTCTATCGTAAAATCGGTATGAAGGTGGTTCAGGCGGGTTCACAGGCGTGGGAGCAACGCCAGTCCACCAGCACGCCAGAGAAACTGGCACTGCTGGTGGCGGCATCGGGTCAGGCGGTTACTTCCCGGGTGGCGCGTCACGCTCCACGCTCAGCTGATCTCTGGCAGCGCCCCGTTTAAGGATCC |
